# Chitosan Mediates Germling Adhesion in *Magnaporthe oryzae* and Is Required for Surface Sensing and Germling Morphogenesis

**DOI:** 10.1371/journal.ppat.1005703

**Published:** 2016-06-17

**Authors:** Ivey A. Geoghegan, Sarah J. Gurr

**Affiliations:** 1 Department of Plant Sciences, University of Oxford, Oxford, United Kingdom; 2 Biosciences, University of Exeter, Exeter, United Kingdom; Wageningen University, NETHERLANDS

## Abstract

The fungal cell wall not only plays a critical role in maintaining cellular integrity, but also forms the interface between fungi and their environment. The composition of the cell wall can therefore influence the interactions of fungi with their physical and biological environments. Chitin, one of the main polysaccharide components of the wall, can be chemically modified by deacetylation. This reaction is catalyzed by a family of enzymes known as chitin deacetylases (CDAs), and results in the formation of chitosan, a polymer of β1,4-glucosamine. Chitosan has previously been shown to accumulate in the cell wall of infection structures in phytopathogenic fungi. Here, it has long been hypothesized to act as a 'stealth' molecule, necessary for full pathogenesis. In this study, we used the crop pathogen and model organism *Magnaporthe oryzae* to test this hypothesis. We first confirmed that chitosan localizes to the germ tube and appressorium, then deleted *CDA* genes on the basis of their elevated transcript levels during appressorium differentiation. Germlings of the deletion strains showed loss of chitin deacetylation, and were compromised in their ability to adhere and form appressoria on artificial hydrophobic surfaces. Surprisingly, the addition of exogenous chitosan fully restored germling adhesion and appressorium development. Despite the lack of appressorium development on artificial surfaces, pathogenicity was unaffected in the mutant strains. Further analyses demonstrated that cuticular waxes are sufficient to over-ride the requirement for chitosan during appressorium development on the plant surface. Thus, chitosan does not have a role as a 'stealth' molecule, but instead mediates the adhesion of germlings to surfaces, thereby allowing the perception of the physical stimuli necessary to promote appressorium development. This study thus reveals a novel role for chitosan in phytopathogenic fungi, and gives further insight into the mechanisms governing appressorium development in *M*.*oryzae*.

## Author Summary

*Magnaporthe oryzae* is a filamentous fungal pathogen which causes devastating crop losses in rice. Successful invasion of the host is dependent upon the ability of the fungus to remain undetected by the innate immune system of the plant, which recognizes conserved components of the fungal cell wall, such as chitin. Previous studies have demonstrated that infection-related changes in cell wall composition are necessary to allow the fungus to remain undetected during infection. One such change that has long been hypothesized to have a role as a 'stealth mechanism' is the deacetylation of the polysaccharide chitin by enzymes known as chitin deacetylases. The deacetylation of chitin produces a polysaccharide known as chitosan, which has previously been shown to accumulate specifically on infection structures in plant pathogenic fungi. However, in this study, we show that germling-localized chitosan is not required for pathogenicity, arguing against a role as a 'stealth mechanism' at this stage. Instead, chitosan is required for the development of the appressorium, a critical fungal infection structure required for the penetration of plant cells. This requirement can be attributed to chitosan mediating the adhesion of germlings to surfaces, which is required for the perception of physical stimuli.

## Introduction

All fungal cells are encased within a cell wall. This complex and dynamic structural barrier is composed of interwoven polysaccharides and proteins. Indeed, the polysaccharide moiety makes up the majority of the fungal wall, being comprised of chitin (a polymer of β1,4-*N*-acetylglucosamine), β/α1,3-glucans and mannans [1]. There is, however, considerable variation in the proportion of these wall components between different cell-types and between fungal species [1]. The cell wall plays an essential role in maintaining cellular integrity in the face of the challenging and varied environmental conditions to which fungi are exposed. Indeed, the cell wall plays a far greater role than simply being the extracellular “coat of armour” [2]. Firstly, morphogenesis is heavily influenced by cell wall composition, and, conversely, localized changes in composition allow fungal cells to undergo morphogenesis, whilst maintaining cellular integrity [3]. This is illustrated by many studies, where chemical or genetic perturbation of cell wall composition has led to gross defects in fungal growth and morphogenesis (reviewed in [1, 4]). Secondly, the cell wall forms the interface between the fungal cell and its immediate environment. As a result, the structure and composition of the cell wall influences both physical and biological interactions which occur over the life-cycle of the fungus, as, for example, during host infection [2].

The hemibiotrophic fungus *Magnaporthe* oryzae causes significant losses of rice [5]. It is thus a notable pathogen but is also considered as a model organism to study appressorium formation in pathogenic fungi [6]. Disease occurs when three–celled asexual conidia land on the host, differentiate a short germ tube and thence an infection structure (the appressorium). This developmental progression occurs in response to hard, hydrophobic surface and following the perception of host-derived surface chemistries, such as cutin [7]. The maturing appressorium generates a considerable turgor pressure, a penetration peg emerges and penetrates the leaf cuticle [8]. Subsequently, invasive hyphae ramify though the host. Throughout this process the fungal wall undergoes extensive remodelling during rapid growth [9].

For successful infection *M*. *oryzae* must remain undetected by its host: but how does the fungus do this? Plants readily detect key molecular signatures of fungal cells, that is, Pathogen Associated Molecular Patterns (PAMPs). Fungal cell wall polysaccharides in particular represent a major source of PAMPs [10]. The best characterized of these are chitin oligomers, which are released from the fungal wall either as a result of endogenous cell wall remodelling, or due to the action of plant chitinases. Chitin oligomers are recognized in rice plants by the Pattern Recognition Receptor (PRR) CEBiP (Chitin Elicitor Binding Protein) [11]. Binding of chitin oligomers to CEBiP induces its dimerization and, in association with CERK1 (Chitin Elicitor Receptor Kinase-1), results in phosphorylation and activation of CEBiP [12]. This triggers PAMP Triggered Immunity (PTI) [13]. This response includes callose deposition, the production of degradative enzymes (chitinases and glucanases), and a burst of reactive oxygen species [14, 15]—all of which serve to restrict or kill the invading fungus. Phytopathogenic fungi have evolved a number of strategies to avoid triggering PTI. The first is by secretion of chitin-binding effectors, which compete with the chitin receptors to bind the oligomers, and thus enable full pathogenesis, as in *Cladosporium fulvum* and *M*.*oryzae* [16, 17]. The second strategy is to change cell wall composition to either alter identity, or to mask the presence of the PAMPs. For example, during infection-related development, *M*.*oryzae* synthesises α1,3-glucan as a component of the cell walls of germ tubes, appressoria and invasive hyphae [9]. Deletion of the sole gene encoding α1,3-glucan synthase (*AGS1*) results in enhanced susceptibility to enzymatic degradation, the triggering of PTI and thus loss of pathogenicity [18]. This suggests that α1,3-glucan acts as a 'stealth' mechanism, cloaking the fungus from recognition by the plant. We postulated that chitin deacetylation may play a similar role—as it constitutes another infection-related cell wall alteration, observed to occur in a number of fungal plant pathogens, including *Uromyces fabae*, *Colletotrichum graminicola*, *Puccinia graminis* and *M*.*oryzae* [9, 19].

Chitin deacetylation is catalyzed by a family of conserved carbohydrate esterase enzymes (CE-4 family) known as chitin deacetylases (CDAs) (E.C. 3.5.1.41). The removal of acetyl groups from *N*-acetylglucosamine residues of chitin results in the formation of chitosan, typically a heterogeneous polymer of β1,4-glucosamine and β1,4-*N*-acetylglucosamine [20]. The deacetylation of chitin confers two hypothetical benefits upon the invading fungus. Firstly, unlike chitin, chitosan cannot cause activation of CEBiP [12]. In addition, although various defence responses have been observed in plants treated with chitosan (reviewed in [21], it is unclear whether chitosan is as effective as chitin in this respect. Secondly, chitin deacetylation likely provides protection from plant chitinases, since chitosan is a poor substrate for these enzymes [22]. However, such roles for chitosan, acting as either a protectant or as a 'stealth' mechanism are not yet supported by direct experimental evidence. Moreover, chitin deacetylases genes are present in the genomes of all fungi [23]. Thus, chitosan may be a broadly-occurring cell wall component, with roles beyond those we hypothesize for plant pathogens. Indeed, such additional roles may relate to the different physical and chemical properties of chitin and chitosan. Chitin occurs in fungal cell walls as antiparallel chains, forming microfibrils with immense strength and rigidity [24]. However, chitin deacetylation creates primary amine groups, which are largely protonated and charged at physiological pH. Consequently, chitosan is polycationic, more hydrophilic and an amorphous polysaccharide, in stark contrast to its parent polymer chitin. Thus, deacetylation could have profound consequences on the structure and physical properties of the fungal cell wall, which, in turn, will impact on fungal growth and morphogenesis.

There is, however, little published data defining the role(s) of chitosan in fungal cell walls *per se*. The role of chitin deacetylation was first described in *S*.*cerevisiae* [25] Here, two functionally redundant CDAs deacetylate chitin specifically in the ascospore cell wall—the double knockout mutant Δ*cda1*Δ*cda2* strain results in complete loss of chitosan from the ascospore wall. Whilst spore viability was unaffected, spores of the Δ*cda1*Δ*cda2* strain were more susceptible to treatment with lytic enzymes, ether and heat shock, suggesting possible disorganization and increased permeability of the wall [25, 26]. Further experimentation showed that the outer dityrosine layer was absent in *CDA* deletion strains [27]. It is hypothesized that the dityrosine is cross-linked to the amine groups of the chitosan, thereby creating a rigid and impermeable ascospore cell wall [28]. Similarly, chitosan was shown to be a component of spore wall in *Ashbya gossypii*—deletion of the single *CDA* resulted in a complete loss of sporulation, suggesting that chitosan is required for spore development [29]. In the Basidiomycete fungus *Cryptococcus neoformans*, chitosan is a major component of the vegetative cell wall [30]. Deletion of 3 of the 4 *CDA* genes (*CDA1-3*) abolished chitosan synthesis [31]—these triple deletion strains were hypersusceptible to various cell wall perturbants, showed attenuated virulence and “leaked” melanin from their cell walls [32] -Chitosan is thus required for cell wall integrity in this fungus..

Deacetylation of chitin may play critical roles in fungal development, integrity and pathogenesis. Thus the two central hypotheses regarding its role are: i) chitin deacetylation is important for cellular development and morphogenesis and ii) chitosan acts mechanisitically as a 'stealth molecule' during plant infection in pathogenic fungi. Appressorium development in *M*.*oryzae* represents an amenable and relevant system to test these hypotheses. Firstly, chitosan is known to be a component of the germ tube and appressorium cell wall [9]. Secondly, protective cell wall components (α1,3-glucan) in appressoria are required for pathogenicity [18]; this may hold true for chitosan. Lastly, appressorium development likely requires increased cell wall flexibility—chitin deacetylation may enable such plasticity. Indeed, this may be the most likely scenario as deletion of a putative chitin deacetylase, *CBP1* resulted in defective appressorium formation on artificial surfaces [33]. Yet, deletion of *CBP1* also prevented hyphopodium and pre-invasive hypha formation on artificial surfaces (but not root surfaces), suggesting that chitosan plays a role in multiple infection-related cellular differentiation events [34]. However, chitosan synthesis was not consistently reduced in the *cbp1* mutant [34] and neither was the chitin deacetylase activity of Cbp1 proven [33] and so the role of chitin deacetylation remains inconclusive. A comprehensive characterization of chitin deacetylases operating during appressorium development should provide a valuable insight into the roles of chitin deacetylation during infection in plant pathogenic fungi.

## Results

### Chitin deacetylation occurs during appressorium development

**Fig 1:**
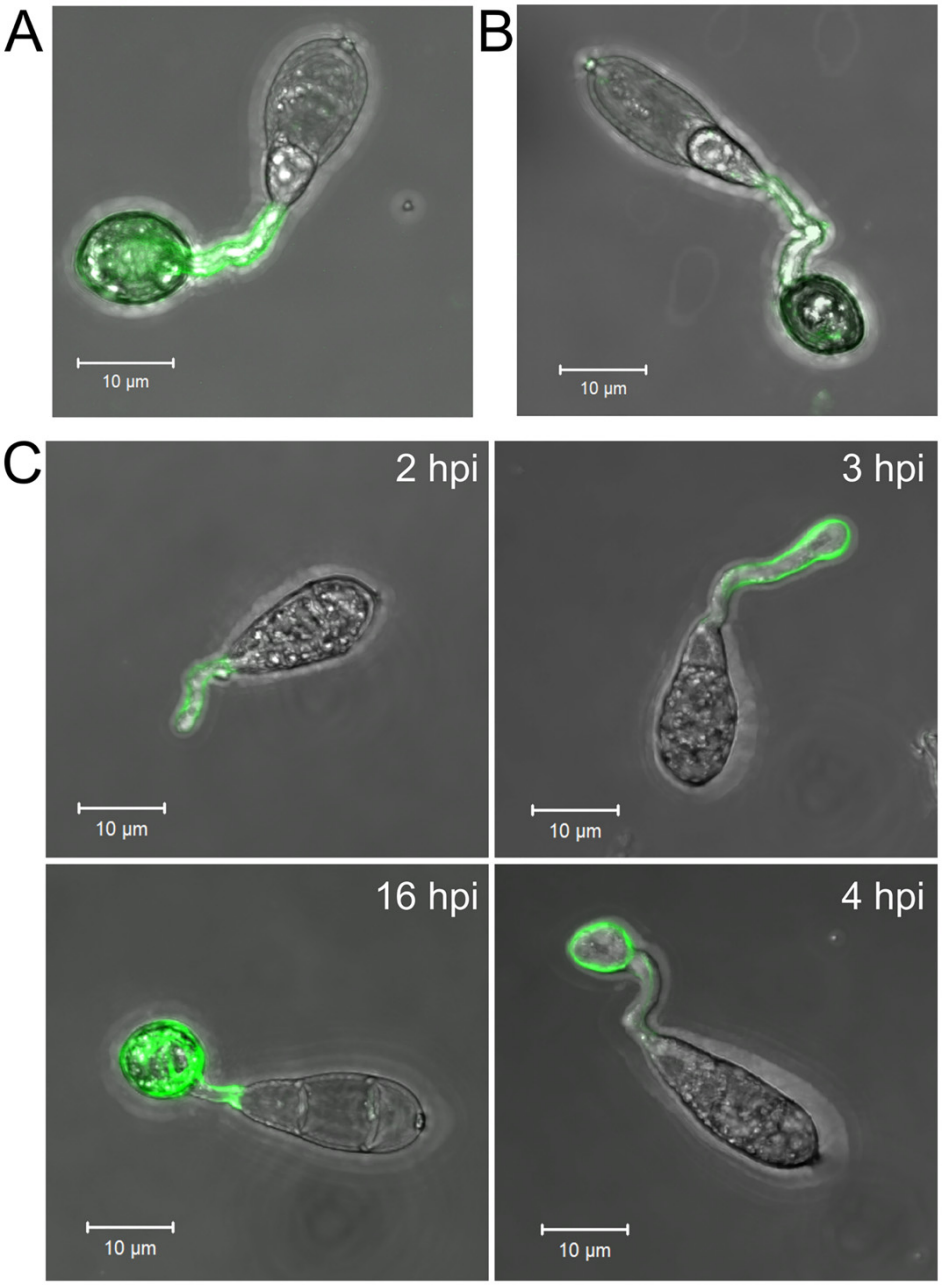
Chitosan is a component of the cell wall in germ tubes and appressoria. Germlings, labelled with **A**) monoclonal anti-chitosan antibody mAbG7, **B**) polyclonal anti-chitosan antibody (staining with antibodies was performed at 16 hpi) and **C**) The anti-chitosan probe OGA488 which localized chitosan in live cells at different stages of appressorium development (2–16 hpi, as indicated). Germlings were inoculated onto a hydrophobic glass surface (inductive to appressorium formation). Scale bars: 10 μm.

The localization of chitosan during appressorium development in *M*.*oryzae* has previously been investigated using Eosin Y (9). Whilst this dye binds to chitosan, via an electrostatic interaction, the specificity of this interaction has not been directly proven. We sought an alternative, more specific method, that is by immune-detection, with mAbG7, a monoclonal anti-chitosan antibody [35], and a polyclonal anti-chitosan antibody [19]. These antibodies revealed that chitosan localizes to the cell wall of the germ tube and the appressorium (Fig 1A & 1B), and is consistent with previous observations made with Eosin Y [9]. However, we wished to determine the precise timing of chitin deacetylation during appressorium development in order to assess whether it is, indeed, required for this process. To do this, we invoked use of the probe OGA488. This recently developed and highly specific probe [36] stains chitosan rapidly (within 15 minutes), and is thus ideally-suited to studying germlings *in vivo* at different stages of morphogenesis. Using this probe, germling development was tracked on an artificial hydrophobic surface inductive to appressorium formation [37]. After 1–2 hpi (hours post inoculation) the germ tube was weakly labelled, labelling strengthened upon onset of germ tube hooking (2–3 hpi), and intensified further during appressorium development (4 hpi). At this stage, the entire germ tube and appressorium wall were labelled—this being consistent with the immune-localisation data, (Fig 1C).

### Chitin deacetylases are expressed during appressorium development

**Fig 2:**
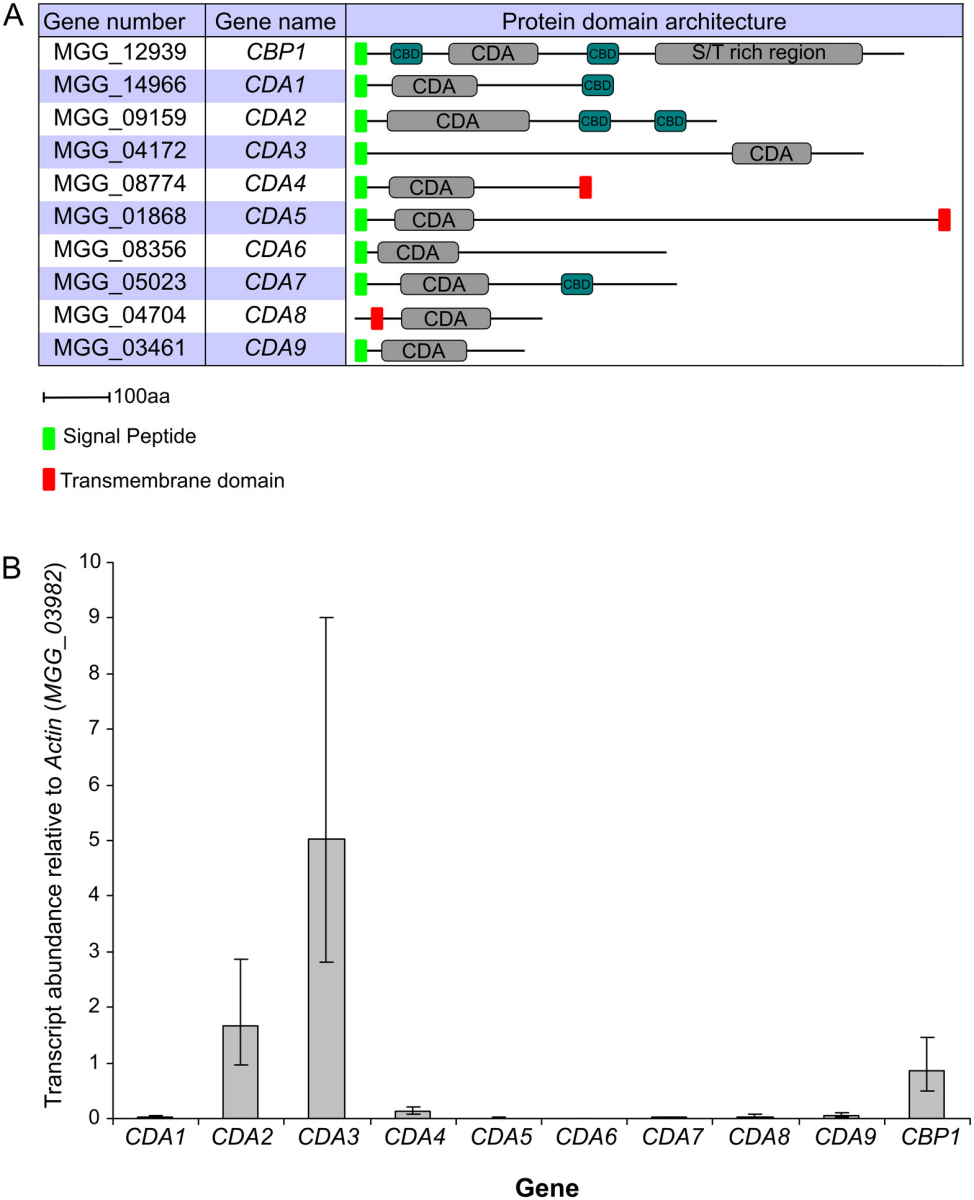
*M*.*oryzae* has 10 putative chitin deacetylases. **A**) Table of the 10 putative chitin deacetylases in *M*.*oryzae*, showing gene numbers, given gene names and protein domain architectures (as defined by Pfam). **B**) Expression of the chitin deacetylases during appressorium development on rice leaves. Leaves of the susceptible cultivar CO-39 were inoculated with conidia of *M*.*oryzae*, and incubated for 5 hr, to induce appressorium development. Expression levels of the *CDA*s were analyzed by qRT-PCR and normalized to *Actin* (*MGG_03982*). Error bars show standard deviation, n = 3.

Next, we asked which of the 10 putative chitin deacetylase genes residing in the *M*.*oryzae* genome (http://www.broadinstitute.org/annotation/genome/magnaporthecomparative/MultiHome.html) are responsible for chitin deacetylation occuring prior to and during appressorium development. The 10 *M*.*oryzae* CDAs carry a polysaccharide/chitin deacetylase domain (Pfam 01522). All, except Cda9, have a predicted N-terminal signal peptide. Putative chitin binding domains (CBD; Pfam 00187) are present in Cbp1, Cda1, Cda2 and Cda7. Cbp1 also has a serine/threonine-rich repeat region at its C-terminus [33]. Cda4, Cda5 and Cda8 have putative single transmembrane domains, suggesting that these proteins localise to the membrane (Fig 2A).

Chitin deacetylation in *Colletotrichum lindemuthianum* is dependant upon a zinc-binding Asp-His-His triad, and four key active site residues: a catalytic base residue (Asp) and a catalytic acid residue (His), which interact with Arg and Asp residues respectively [38, 39]. Sequence alignment using Clustal Omega [40], revealed conservation of the zinc-binding triad and active-site resides in all *M*.*oryzae* CDA sequences, with the exception of a non-catalytic Asp in Cbp1 (MGG_12939) at position 153 of the alignment (S1 Fig).

To determine which of the 10 *CDA*s are expressed during appressorium development, we performed qRT-PCR analysis. Total RNA was extracted from rice leaves inoculated with WT conidia, at 5 hpi. Appressorium development was confirmed at this timepoint by microscopic observation (S2 Fig). Relative quantification of transcript abundances for all 10 *CDA*s revealed that *CDA3* is the most highly expressed, followed by *CDA2* and *CBP1* (Fig 2B). Low levels of expression were observed for the remaining genes. In addition to this, we also analyzed the expression of the *CDA*s during later stages of leaf infection, at 36 hpi. In this case, *CDA1* and *CDA6* showed the highest transcript abundance (S2 Fig). These data align with published transcriptomics datasets [41, 42].

### Chitin deacetylases are required for appressorium development on artificial surfaces

The combined transcriptional profiling data reveals that three particular *CDA* genes are likely involved in chitin deacetylation during appressorium development: *CBP1*, *CDA2*, and *CDA3*. *CBP1* has previously been partially characterized [33], but we wished to further this work, in combination with *CDA2*, and *CDA3* and thus generated single, double and triple deletion strains by a targeted gene replacement approach. All such mutants generated were confirmed by both PCR and by Southern Blot analysis (S3 and S4 Figs). Deletion strains are listed in S1 Table.

**Fig 3:**
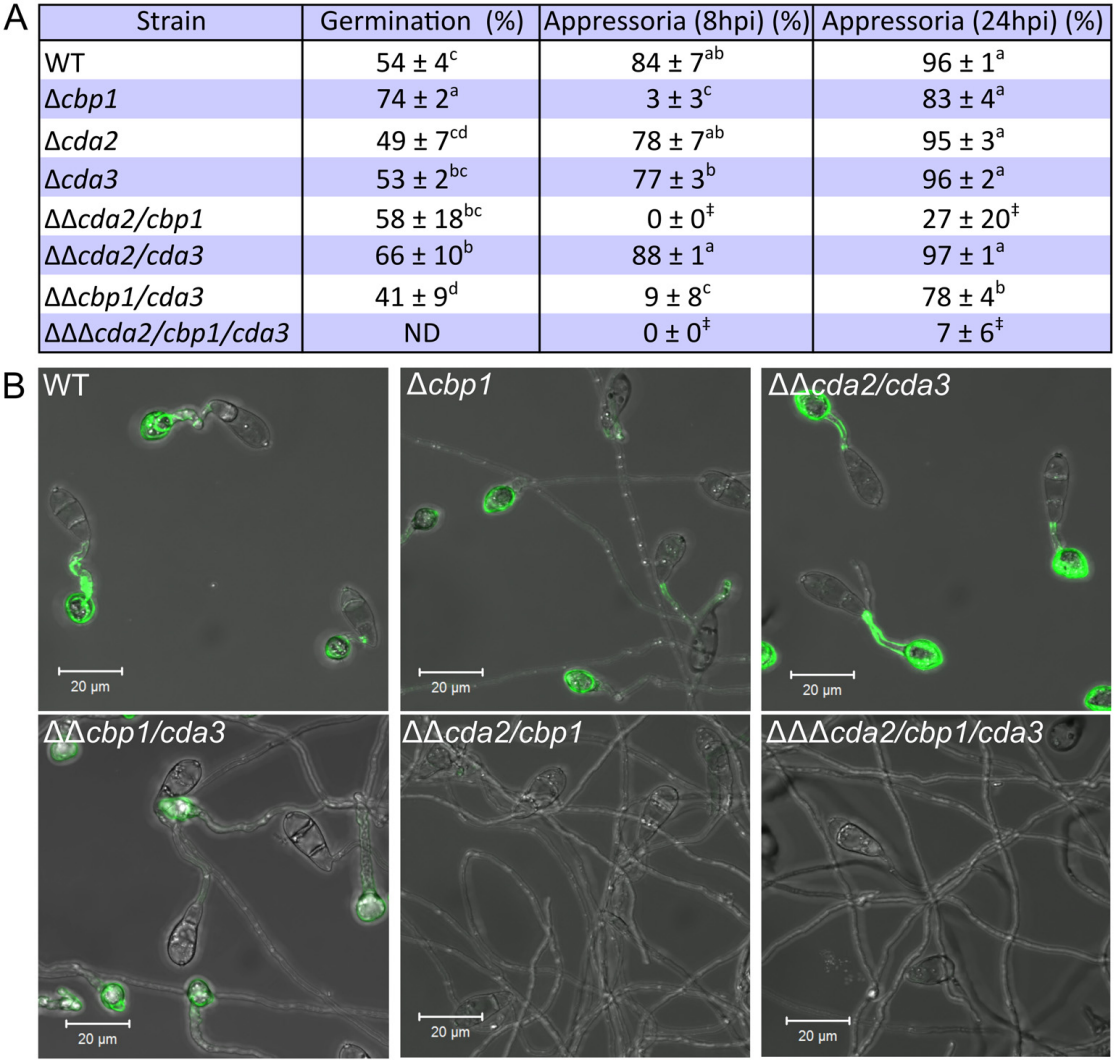
Appressorium development and chitin deacetylation are dramatically reduced in the *CDA* deletion strains. **A)** Table showing percentage conidial germination (at 1 hpi) and percentage of germ tubes forming appressoria (at 8 and 24 hpi) on a hydrophobic glass surface (± SD, n = 3). Germination rates were analyzed using Welch's ANOVA, with Games-Howell post-hoc test. Means not sharing a letter indicate significant differences (p < 0.05). Significantly reduced appressorium formation frequency was observed in certain deletion strains (1-way ANOVA with post-hoc Tukey test. Means not sharing a letter indicate significant differences p < 0.05). ‡: data could not be analyzed using ANOVA, as did not fulfil requirements. **B)** Fluorescent labelling of chitosan on germlings of the *CDA* deletion strains at 16 hpi, showing complete lack of staining in the *cda2/cbp1* and *cda2/cbp1/cda3* strains. Scale bars: 20 μm.

To compare appressorium development, conidia of the mutant and WT strains were inoculated onto an artificial hydrophobic glass surface. In the WT strain, over 80% of conidia developed appressoria after an 8 hr incubation (Fig 3A). However, very few appressoria formed in *cbp1* at 8 hpi (3% ± 3% (SD n = 3))—germ tubes were abnormally elongated and failed to hook at the end distal from the spore (Fig 3B). This is consistent with the previous report [33]. Germination of *cbp1* conidia was significantly higher than the WT strain at 1 hpi (Welch's ANOVA, p < 0.05, Fig 3A) and thus failure to form appressoria is not due to delayed or reduced germination. By 24 hpi, 83% ± 4% of *cbp1* germ tubes formed appressoria (Fig 3A) that were misshapen in appearance (S6 Fig). Appressorium development is therefore both delayed and defective in *cbp1*. In the *cda2*, *cda3* and *cda2/cda3* strains, appressorium development and conidial germination progressed similarly to WT. However, deletion of *CDA2* together with *CBP1* resulted in much more severe defects in appressorium development than observed in the single deletion strain *cbp1*. Germlings of strain *cda2/cbp1* failed to form appressoria by 8 hpi, as in *cbp1*, but extending the incubation period to 24 hpi resulted in only rare occurrences of appressorium development (27% ± 20 of germ tubes), in contrast to *cbp1* (Fig 3A). The germ tubes of *cda2/cbp1* appeared even more elongated (487 ± 42 μm in *cda2/cbp1* compared with 264 ± 37 μm in *cbp1* (S3 Table)), yet remained undifferentiated. This phenotype was also observed when conidia were germinated on other hydrophobic surfaces, including plastic and Parafilm (S5 Fig). *CDA2* and *CBP1* therefore exhibit partial redundancy. Conversely, deletion of *CDA3* had no additive effects on appressorium development under the conditions tested: *cbp1/cda3* demonstrated an identical phenotype to *cbp1*, and *cda2/cbp1/cda3* a similar phenotype to *cda2/cbp1*.

To determine whether chitin deacetylation is reduced in the *cda* mutants, germlings of the deletion strains were stained with OGA488 (Fig 3B). WT germlings developed on an artificial hydrophobic surface showed strong staining of the cell wall in germ tubes and appressoria at 16 hpi, as described previously. Similar staining was observed in *cda2/cda3*. In *cbp1* and *cbp1/cda3*, appressoria labelled with OGA488, but little fluorescence was observed on their elongate germ tubes. However, no chitosan staining was observed in strains *cda2/cbp1* and *cda2/cbp1/cda3*, suggesting complete loss of chitin deacetylation activity, and providing further evidence of functional redundancy between *cbp1* and *cda2*. By 24 hpi a small proportion of germ tubes in strains *cda2/cbp1* and *cda2/cbp1/cda3* formed appressoria. However, no chitosan was detected (S6A Fig), suggesting that it may not be an absolute requirement for appressorium morphogenesis. Yet, the presence of low concentrations of chitosan, or chitosan in cell wall regions inaccessible to the OGA488 probe cannot be completely discounted.

The highly elongate germ tubes observed in strains *cbp1*, *cda2/cbp1* and *cda2/cbp1/cda3* are a curious feature. Calcofluor White staining revealed that the mutant germ tubes are septated, with cross wall distributed along the elongate germ tubes of *cbp1*, *cda2/cbp1* and *cda2/cbp1/cda3* at regular intervals (S6B Fig). Taken together, these data suggest a clear link between the loss of chitin deacetylation, and loss of appressorium development.

### Localization of chitin deacetylases

We created fluorescent protein fusions, to better understand how chitin deacetylation by Cbp1 and Cda2 promotes appressorium development. C-terminal mCherry fusions of *CBP1* and *CDA2* were made, under the control of their respective native promoters, and transformed into their respective deletion background strains. Several independent transformant lines were characterized for each fusion and which exhibited identical patterns of fluorescence (S1 Table).

**Fig 4:**
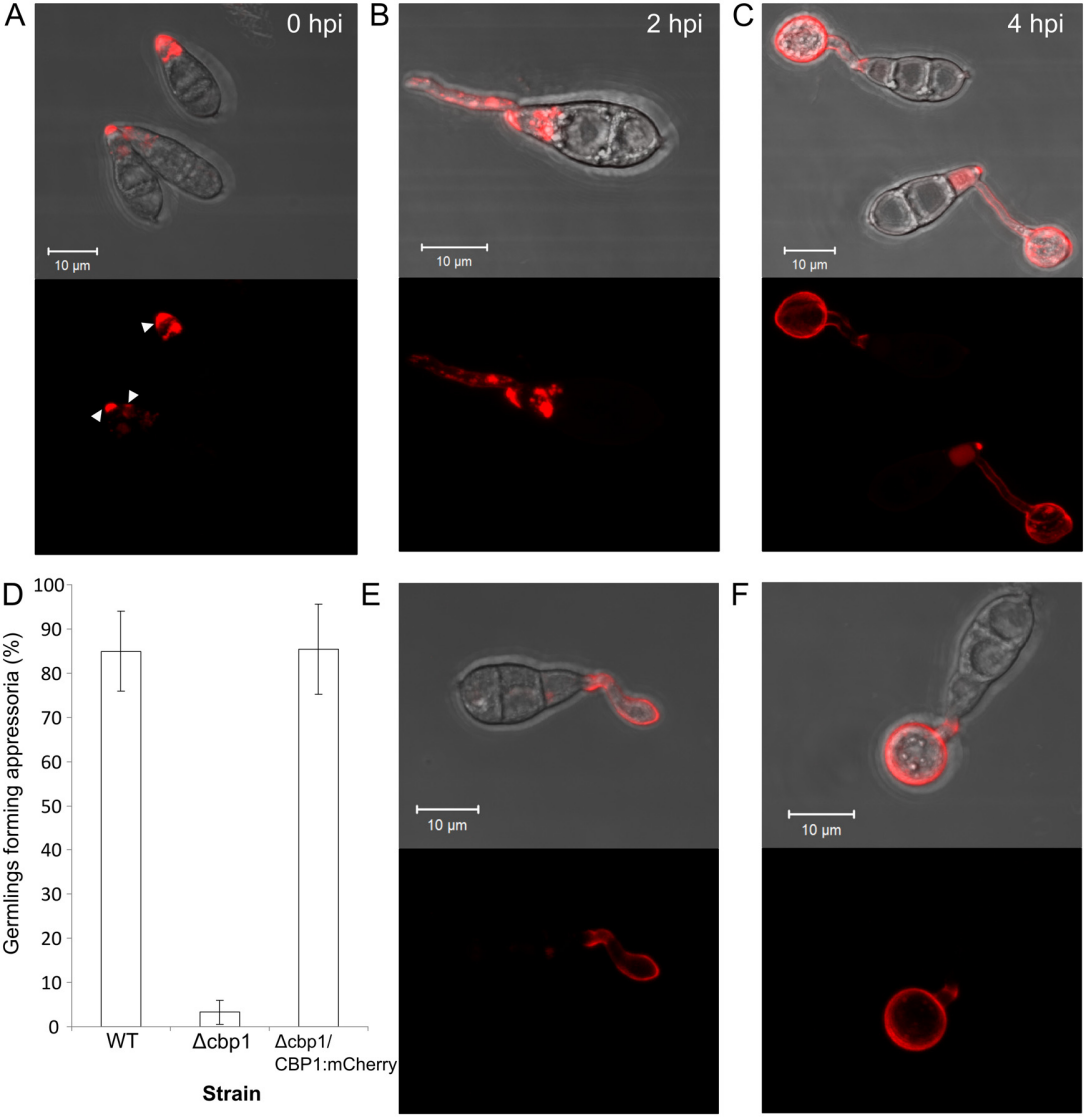
Localization of mCherry tagged Cbp1 and Cda2. Confocal fluorescence microscopy images of germlings expressing Cbp1:mCherry, showing fluorescence in conidia, germ tubes and appressoria at 0, 2 and 4hpi on hydrophobic glass (**A-C**). **D**) Successful complementation of *CBP1*:*mCherry* fusion construct. Appressorium development was fully restored in the complemented strain at 8 hpi. Error bars show SD n = 3. **E** & **F)** Confocal fluorescence microscopy images of germlings expressing Cda2:mCherry at 2 hpi (**E**) and 4 hpi (**F**). Scale bars: 10 μm.

Strains expressing Cbp1:mCherry show fluorescence at all stages of germling morphogenesis (Fig 4A–4C), and demonstrate fully restored appressorium development (Fig 4D), indicating that the Cbp1:mCherry fusion protein is fully functional. Unexpectedly, fluorescence was observed in the cell wall at conidial apices, even in conidia which had not yet been harvested from the mycelium (Fig 4A). Substantial intracellular fluorescence was also observed at this stage, most likely localized to vacuoles. During initial stages of germ tube growth (1–2 hpi), weak fluorescence was observed at the lateral walls of the germ tube, but not the tip (Fig 4B). In addition, small punctae of intracellular fluorescence were sometimes observed along the length of the germ tube. At later stages of appressorium development (3–5 hpi), wall-localized fluorescence became stronger and localized to the entire germ tube and appressorium, although intracellular fluorescence was still apparent in some appressoria (Fig 4C). The appressorial wall remained fluorescent even at later stages (8–16 hpi, S7 Fig), although intensity decreased noticeably.

Similarly to Cbp1:mCherry, strains expressing Cda2:mCherry exhibited fluorescence during appressorium development, but with fluorescence typically appearing after hooking of the germ tube (Fig 4E). Strong fluorescence was also observed during subsequent appressorium formation (Fig 4F), and remained present at later stages (8 hpi), as observed with Cbp1:mCherry (S7 Fig). Unlike Cbp1:mCherry however, fluorescence was only very rarely observed intracellularly. Since the *cda2* strain was apparently identical to the WT strain, functionality of the fusion protein could not be determined in the complemented strain. Attempts to create a Cda3:eGFP fusion protein did not yield fluorescent transformants, suggesting that the fusion protein is unstable.

### Exogenous chitosan can rescue *cda* mutants

**Fig 5:**
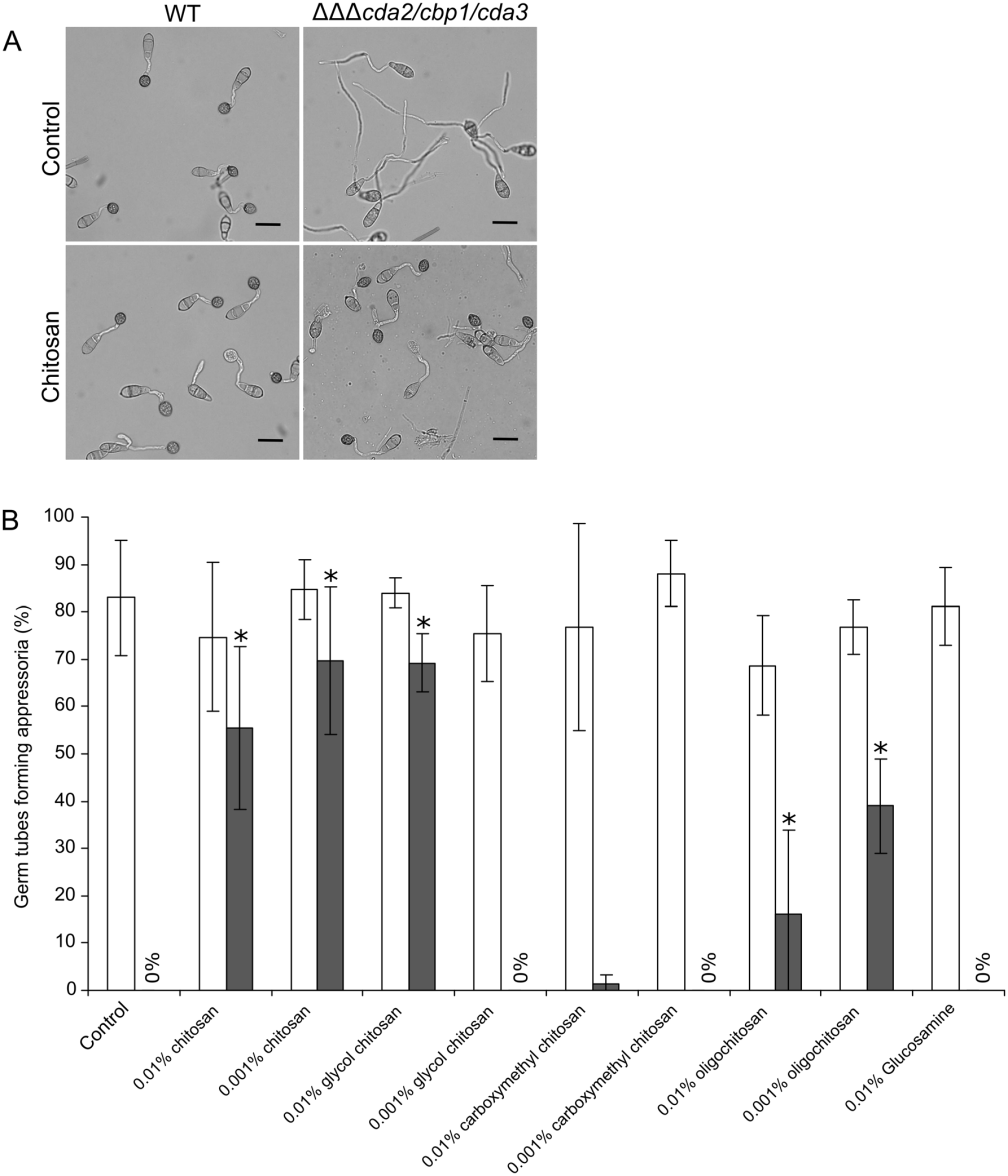
Exogenous chitosan can rescue appressorium development in *CDA* deletion strains. **A**) Rescue of appressorium development in the *cda2/cbp1/cda3* deletion strain with exogenously applied chitosan (0.01% w/v). Pictures taken at 8 hpi, scale bars: 20 μm. **B**) Comparison of appressorium development in WT (white bars) and *cda2/cbp1/cda3* deletion strain (grey bars) in the presence of different derivatives and formulations of chitosan (shown in S8 Fig). Conidia were germinated on an artificial inductive surface for 8 hr in the presence of different types of chitosan at a final concentration of 0.01% or 0.001% (w/v), and mature appressoria were counted. Significant increases in appressorium development were observed in the *cda2/cbp1/cda3* deletion strain (Asterisks, Student's T-test, p < 0.05). Error bars show SD, n = 3. DDA: degree of deacetylation.

The defects in appressorium development observed upon deletion of *CDA* genes was similar to that resulting from deletion of the hydrophobin gene *MPG1* [43]. It was reported that appressorium development could be rescued in the *mpg1* mutant by co-inoculation with WT conidia, suggesting that Mpg1 could act in *trans* [44]. To test whether chitosan could rescue the *cda* mutants, exogenous chitosan was added to conidia during germination on an artificial hydrophobic surface. Addition of 0.01% (w/v) or 0.001% (w/v) chitosan, surprisingly, restored appressorium development in all *cda* deletion strains (Fig 5A & 5B). Between 70–85% of *cbp1*, *cda2/cbp1* and *cbp1/cda3* conidia formed appressoria after an 8hr incubation in the presence of 0.01% or 0.001% chitosan, compared with 0–10% in the control treatment (water). In the triple deletion strain (*cda2/cbp1/cda3*) rescue was slightly lower (55% ± 17 (SD, n = 3) and 70% ± 16 (SD, n = 3) appressoria at 8 hpi for 0.01% and 0.001% chitosan, respectively. In the WT strain, 70–85% of conidia formed appressoria in all treatments. To further investigate this, different derivatives and formulations of chitosan were tested for their ability to restore appressorium development in the deletion strains (structures are shown in S8 Fig). Glycol chitosan, a soluble derivative of chitosan restored appressorium development in *cda2/cbp1/cda3* at a concentration of 0.01% but not 0.001%. Carboxymethylchitosan (in which chitosan has been modified by the addition of an anionic carboxymethyl group) did not restore appressorium formation at either concentration. Lastly, the addition of chitosan in oligomeric form (oligochitosan, with a degree of polymerization of 22–33 residues) partially restored appressorium formation at 8 hpi, whereas glucosamine showed no restorative effects at all. Taken together, these data suggest that the cationic nature of exogenous chitosan is the most important factor determining rescue of the mutant phenotype, with steric factors also having some influence. Polymer length appears to be of little importance, given the broad ranges capable of restoring appressorium development.

**Fig 6:**
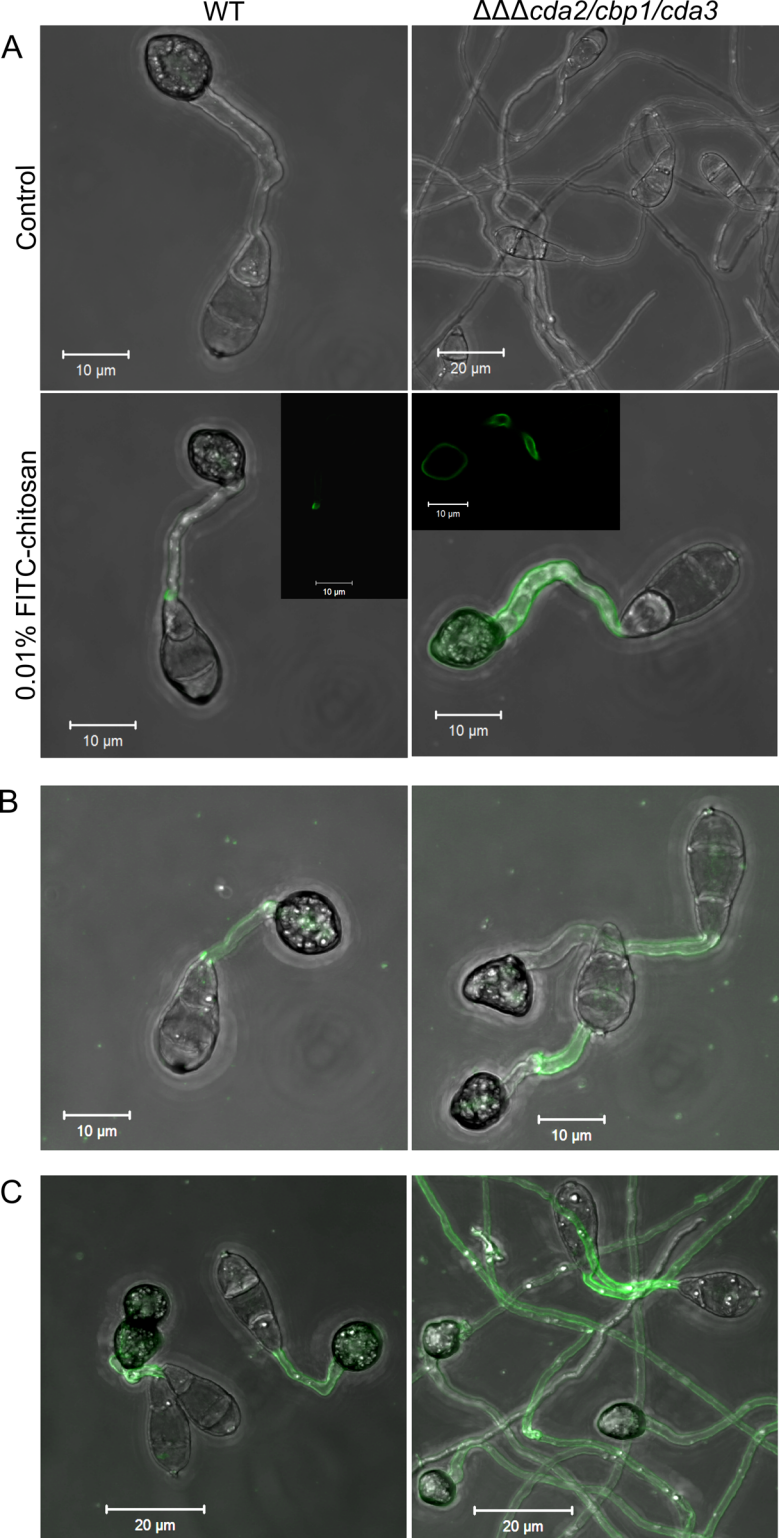
Fluorescently labelled chitosan is associated with the cell wall of the *CDA* deletion strains. **A**) Rescue of appressorium development in the *cda2/cbp1/cda3* deletion strain using exogenous, fluorescently-labelled chitosan, following a 16 hr incubation, showing clear localization to the cell wall of the germ tube and appressorium (single z-section insert). **B**) Rescue of appressorium development after a 2 hr incubation with FITC-chitosan, followed by washing and a further 16 hr incubation, showing germ tube-localized FITC-chitosan. **C**) Rescue of appressorium development after addition of FITC-chitosan at 16 hpi, and incubation for a further 8 hr. Scale bars: as shown in pictures.

To investigate how exogenous chitosan restores appressorium development in the *cda* mutants, fluorescently-labelled chitosan was used. FITC-chitosan, synthesized according to Qaqish et al [45], was added to conidia of the WT and *cda2/cbp1/cda3* strains on an artificial hydrophobic surface. After 16 hr incubation, the FITC-chitosan was removed and the germlings imaged by confocal microscopy. The FITC-chitosan restored appressorium development in *cda2/cbp1/cda3*, and clear localization to the cell wall of germ tubes and appressoria (Fig 6A). In the WT strain, weak fluorescence was occasionally observed in the germ tubes. Exogenous chitosan is therefore associated with the cell wall of the deletion strains.

Chitosan localizes to the germ tube prior to appressorium development (Fig 1C). To determine whether this germ tube-localized chitosan is sufficient to induce appressorium development, FITC-chitosan was added to germinating conidia of WT or *cda2/cbp1/cda3* strains for 2 hr only, before being washed off, and the germlings left to develop for a further 16 hr. In this way, chitosan was only present during the initial germ tube stage of germling morphogenesis, and was removed before appressorium development occurred. Appressorium development was restored in the deletion strain by this short incubation with FITC-chitosan. Here, fluorescence was only observed in the germ tube, and not the appressoria of *cda2/cbp1/cda3* germlings, with similar localization in the WT (Fig 6B). Thus germ tube-localized chitosan seems sufficient to induce appressorium development.

Lastly, to investigate whether chitosan restores appressorium development at later stages of germling morphogenesis, conidia were incubated for 16 hr, FITC-chitosan added, and the conidia incubated for a further 8 hr. Again, appressorium development was restored in the *cda2/cbp1/cda3* strain, although here the germ tubes were highly-elongate (Fig 6C). Fluorescence was observed along the entire length of these elongate germ tubes, but appeared more intense at regions proximal to its point of emergence from the conidium. In this case, WT germlings also exhibited weak fluorescence in both germ tubes and appressoria.

### Pathogenicity of the *cda* mutants

**Fig 7:**
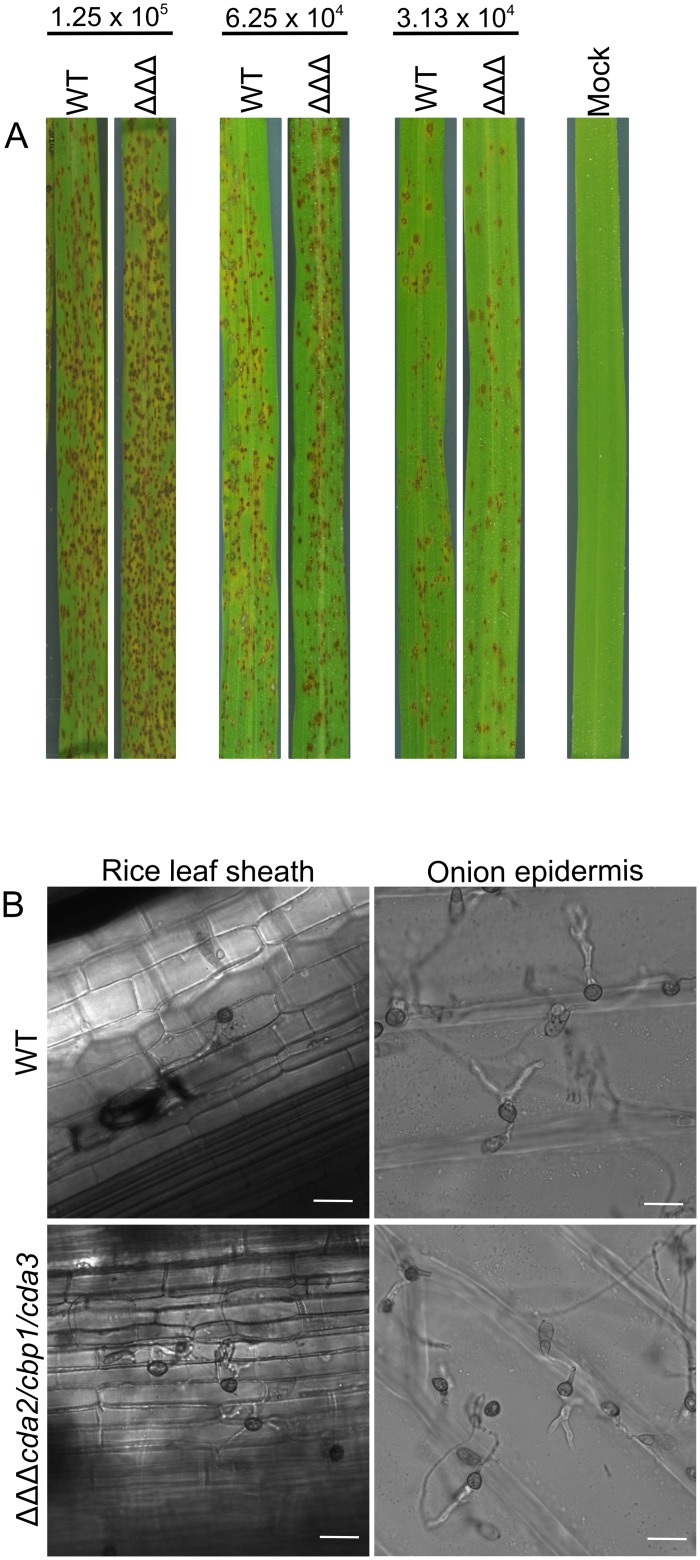
Pathogenicity is unaffected in the *cda* mutants. **A**) Pathogenicity of the WT and *cda2/cbp1/cda3* strains on whole rice plants. Rice plants were inoculated with conidia of the WT or *cda2/cbp1/cda3* strain at 3 different spore concentrations, and incubated for 4 days. The experiment was repeated 3 times, representative leaves are shown. **B**) Appressorium development in the WT and *cda2/cbp1/cda3* strains on rice leaf sheath and on onion epidermis. Restored appressorium development and successful penetration of the plant cells was observed in *cda2/cbp1/cda3*. Scale bars: 20 μm.

Appressorium development is highly defective in *cda* mutants on artificial hydrophobic surfaces, inductive to appressorium formation in the WT strain. To determine whether this results in a reduction in pathogenicity, equal concentrations of conidia of the deletion strains were inoculated onto detached rice leaves. As reported previously for *cbp1* [33] and *cda3* [46], and as expected for *cda2* and *cda2/cda3*, pathogenicity was unaffected in these strains (S9 Fig). Surprisingly however, the double and triple deletion strains were also able to successfully infect detached rice leaves or whole plants, causing similar lesion numbers to the WT strain (Fig 7A and S9 Fig). Previously, appressorium development was found to be restored in the *cbp1* strain on rice leaves [33]. To see if this was also the case in the other deletion strains, rice leaf sheaths were inoculated with conidia of the *cda2/cbp1/cda3* strain, and incubated for 24 hr. This revealed that appressorium development was, indeed, restored upon germination on a leaf surface (Fig 7B). Not only this, but the appressoria of *cda2/cbp1/cda3* were able to penetrate rice cells with similar efficiency to the WT: 67% (±7, (SD) n = 3) of WT appressoria had invasive hyphae at 24 hpi, compared with 47% (±10, (SD) n = 3) of *cda2/cbp1/cda3* appressoria. To see if this effect was specific to the rice leaf surface, the experiment was repeated on onion epidermis. Again, appressorium development was restored in the *cda2/cbp1/cda3* strain and invasive hyphae were observed at 24 hpi (Fig 7B).

### Cuticular waxes can by-pass the requirement for chitosan

**Fig 8:**
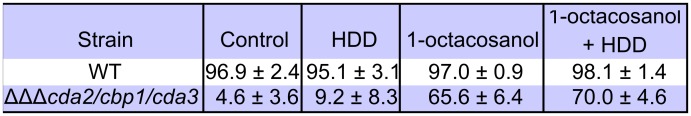
Waxes can partially restore appressorium development in the *cda2/cbp1/cda3* mutant. Percentage of germ tubes demonstrating appressorium development in presence of 1-octacosanol and/or 1,16 hexadecanediol, comparing the WT and *cda2/cbp1/cda3* strain at 24 hpi.

The artificial hydrophobic surface lacks the laminated layer of cutin sandwiched between wax on the rice leaf surface cuticular rice leaf [47]. To test whether wax is sufficient to restore appressorium development in the *cda2/cbp1/cda3* strain, hydrophobic glass coverslips were coated with the wax molecule 1-octacosanol, which has previously been used to induce appressorium development in *M*.*oryzae* [48]. Conidia of strain *cda2/cbp1/cda3* germinated on this surface for 24 hr demonstrated partially restored appressorium development (65.6 ± 6.4% on 1-octacosanol compared with 4.6 ± 3.6% on the control surface) (Fig 8). However, germ tubes of the triple deletion strain remained extremely elongated (S10 Fig).

Cutin monomers are known to induce appressorium development in *M*.*oryzae* [7], To determine whether cutin restores appressorium development in the *cda* mutants, conidia of *cda2/cbp1/cda3* were germinated on an artificial hydrophobic glass surface, in the presence of 1,16-hexadecanediol (HDD; cutin monomer) either on its own, or in combination with 1-octacosanol. HDD did not induce appressorium development in germlings of the *cda2/cbp1/cda3* strain, and no synergistic effect was observed when HDD was used in combination with 1-octacosanol (Student's T-test, no significant difference at p < 0.05, compared with 1-octasosanol treatment alone) (Fig 8 and S10 Fig).

### Chitosan is not required for appressorium development on plant surfaces

**Fig 9:**
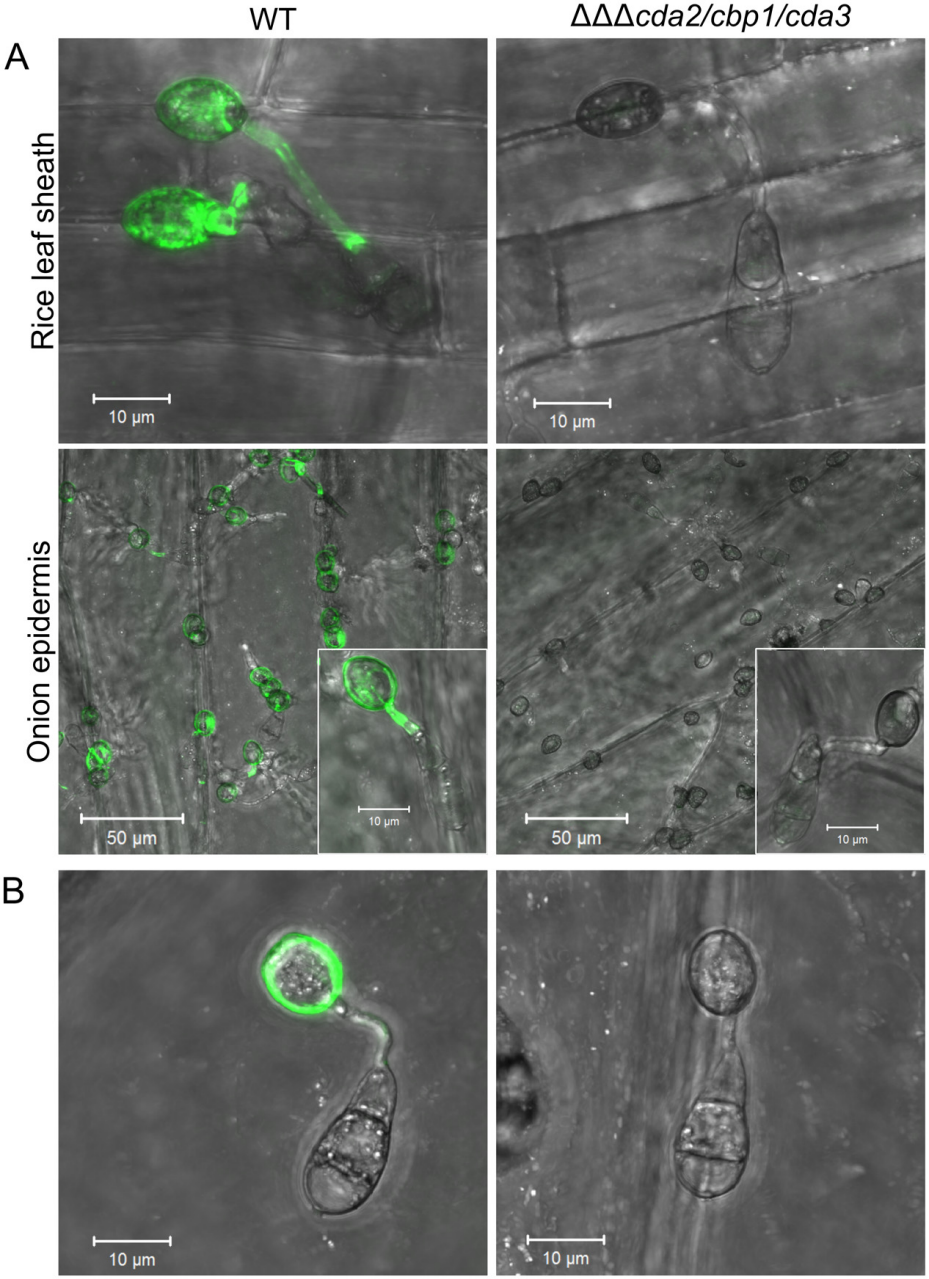
Restored pathogenic development on plant surfaces is not accompanied by restored chitin deacetylation. Fluorescent labelling of chitosan with OGA488 in WT and *cda2/cbp1/cda3* germlings germinated on **A**) rice leaf sheaths or onion epidermis for 24 hr, showing complete absence of chitosan in the *cda2/cbp1/cda3* strain. **B**) Fluorescent labelling of chitosan in WT and *cda2/cbp1/cda3* germlings germinated on onion epidermis for 4 hr, showing absence of chitosan in unmelanized appressoria of the deletion strain. Scale bars: as stated in pictures.

The rescue of appressorium development in the *cda2/cbp1/cda3* strain on plant surfaces is perplexing. We hypothesized that chemical factors present on the leaf surface, such as wax, trigger upregulation of additional, functionally redundant chitin deacetylases, and restore chitin deacetylation. To test this, conidia of the *cda2/cbp1/cda3* and WT strains were germinated on rice leaf sheaths or onion epidermis', and, subsequently stained with OGA488. Chitosan remained completely undetectable in the triple deletion strain, whilst strong labelling of germ tubes and appressoria was observed in the WT (Fig 9A). This suggests that restoration of appressorium development in *cda2/cbp1/cda3* on plant surfaces is not due to the upregulation of redundant chitin deacetylases. Chitosan is thus not an absolute requirement for appressorium development; instead, this requirement appears to be surface dependant.

The appressorial cell wall is impregnated with an impermeable layer of melanin. This may mask detection of chitosan in the appressoria of *cda2/cbp1/cda3*. Mutant strain germlings were also stained with OGA488 at 4 hpi, prior to melanization of the appressoria. However, no OGA488 staining was detected in unmelanized *cda2/cbp1/cda3* germlings, suggesting the melanin does not prevent the detection of chitosan (Fig 9B).

### Chemical inducers can restore appressorium development in *cda* mutants

**Fig 10:**
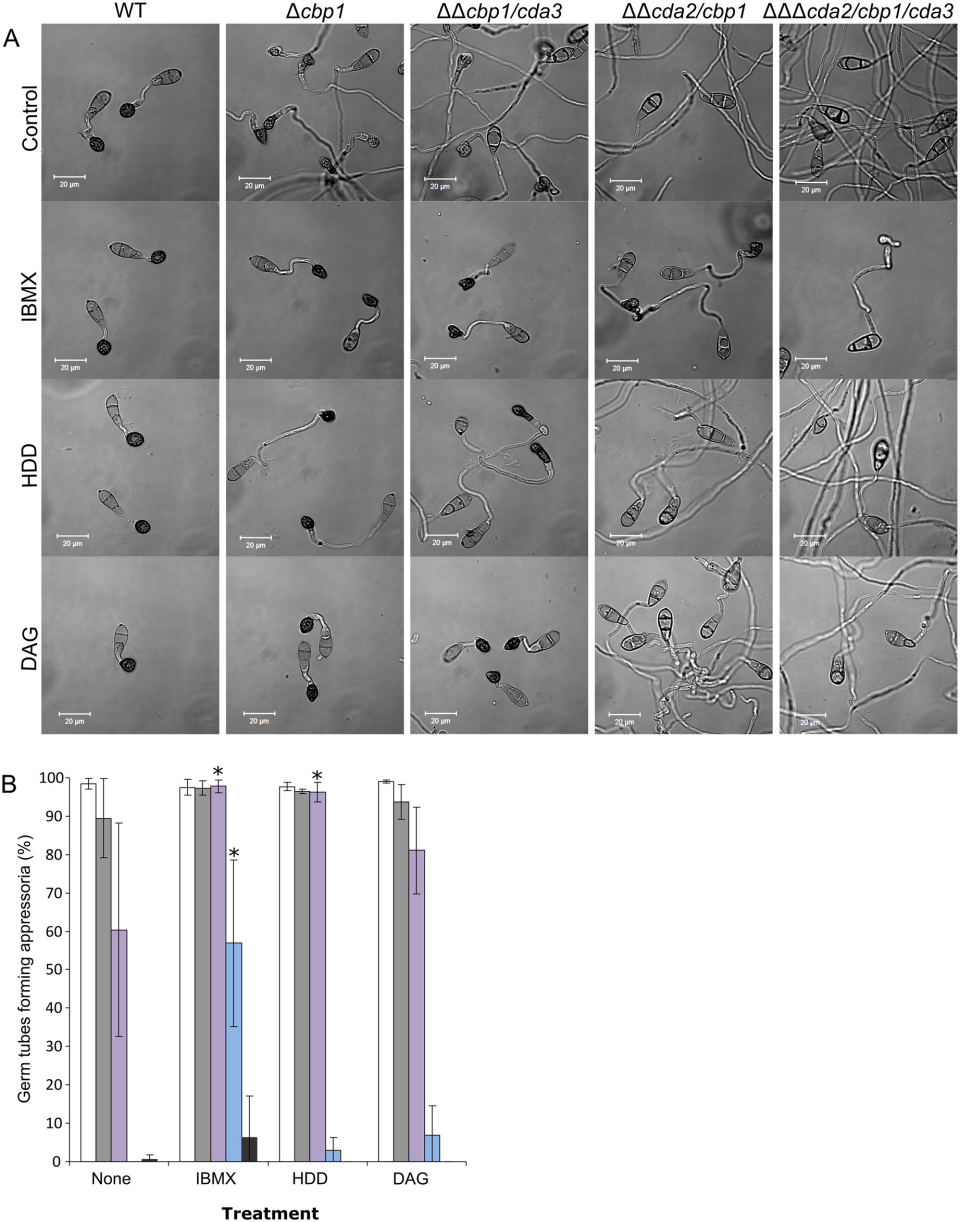
The *CDA* deletion strains show distinct sensitivity to chemical inducers of appressorium development. **A**) Micrographs of germlings at 16 hpi in presence or absence IBMX, 1,16 hexadecanediol (HDD), or diacylglycerol (DAG), showing drastically reduced inductive effects in *cda2/cbp1* and *cda2/cbp1/cda3* strains. Scale bars: 20 μm. **B**) Quantification of appressorium development, as visualized in **A**, WT: White bars, *cbp1*: grey bars, *cbp1/cda*: purple bars, *cda2/cbp1*: blue bars and *cda2/cbp1/cda3*: dark grey bars. Error bars show SD, n = 3. Significant differences in appressorium development were observed upon treatment with inducers in the *cbp1/cda3* and *cda2/cbp1* strains (asterisks, Student's T-test p < 0.05).

Appressorium development in *M*.*oryzae* is controlled by a number of regulatory pathways, which have been shown to respond to the physical and chemical cues present on the leaf surface [49]. In order to determine if there is interplay between chitosan and these particular relays, appressorium development assays were performed in the presence of chemical inducers of these pathways. Previously, appressorium development in *cbp1* has been shown to be restored by the addition of cAMP, 1,16 hexadecanediol or diacylglycerol [33]. We tested the *cda* mutant strains by germinating conidia on a hydrophobic glass surface in the presence of 2.5 mM IBMX (a phosphodiesterase inhibitor which increases intracellular levels of cAMP), 200 mM 1,16 hexadecanediol (HDD, a cutin monomer) or 58 mM diacylglycerol (DAG, an activator of protein kinase C). Consistent with the previous report, all treatments restored appressorium development in *cbp1* (Fig 10). Although the proportion of germ tubes forming appressoria was unchanged in this case (since observations were made at 16 hpi), germling morphology of *cbp1* was now similar to WT, as evidenced by a significant reduction in germ tube lengths (Mann-Whitney U-test, p < 0.001 S3 Table). *cbp1/cda3* showed a similar profile of sensitivity to the chemical inducers as *cbp1*. Intriguingly however, *cda2/cbp1* was considerably less affected by all three chemical inducers: neither HDD or DAG caused a significant increase in appressorium development in this strain (Student's T-test, p < 0.05), whilst IBMX effected only a partial rescue towards the WT phenotype. In *cda2/cbp1/cda3*, IBMX treatment led to a small proportion of germ tubes forming aberrant appressoria by 16 hpi, although this increase was non-significant (Student's T-test, p < 0.05). Rescue of *cbp1* by HDD and DAG is therefore dependent upon *CDA2*, and rescue by IBMX is dependent upon both *CDA2* and *CDA3*.

**Fig 11:**
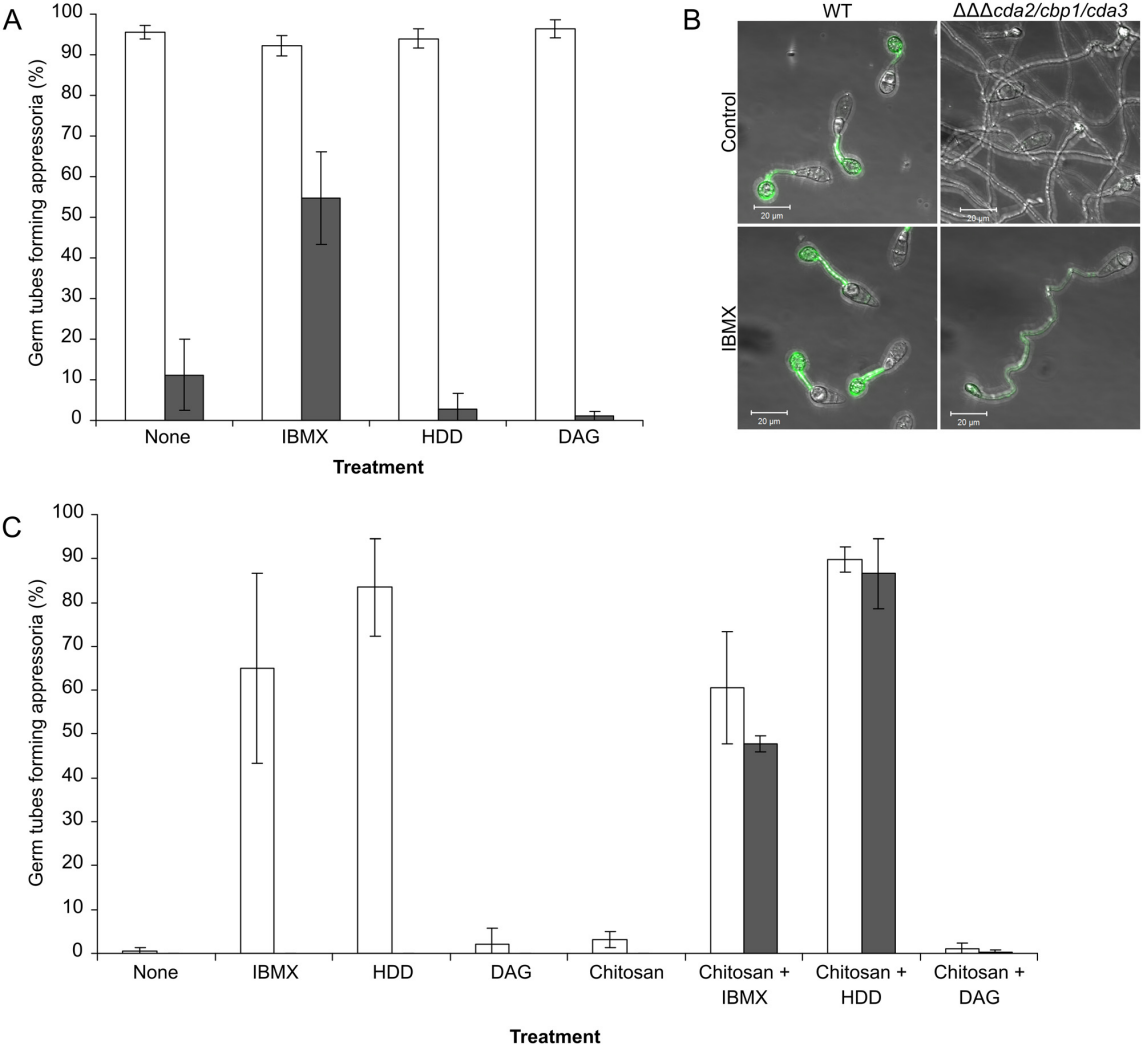
Combinations of chemical inducers are required to restore appressorium development in *cda2/cbp1/cda3*. Induction of appressorium development in the *cda2/cbp1/cda3* strain by chemical inducers, on **A**) hydrophobic and **C**) hydrophilic glass surfaces. Appressorium development could be partially restored in the triple deletion strain (grey bars) after a 24 hr incubation with IBMX, although chitosan staining of these germlings (**B**) showed that the appressorium development was associated with an increase in chitosan in the germlings of the *cda2/cbp1/cda3* mutant. **C**) Appressorium development on hydrophilic surfaces could be restored to levels similar to the WT strain by combinations of 0.01% chitosan (w/v) and 2.5mM IBMX or with 200 μm 1,16 hexadecanediol (HDD). White bars: WT, grey bars: *cda2/cbp1/cda3*. Error bars: SD, n = 3.

Hydrophobicity is one of the key physical cues inducing appressorium development, and is perceived in a cAMP-dependant manner [50]. In order to further examine the relationship between hydrophobic surface sensing, chitosan and the chemical inducers, germling differentiation assays, with the WT and mutant strain *cda2/cbp1/cda3*, were performed on both hydrophobic and a hydrophilic glass surfaces. At 24 hpi on the hydrophobic surface, the response of *cda2/cbp1/cda3* germlings to the inducers was much the same as at 16 hpi, although the proportion of appressoria formed with IBMX was higher (Fig 11A). As with the appressoria formed on the plant leaf surface, it was important to determine whether this rescue could be explained by restored chitin deacetylation. IBMX-treated germlings of the WT and *cda2/cbp1/cda3* strains were stained with OGA488 at 24 hpi. This revealed that chitosan was indeed present on the germ tubes and appressoria of the triple deletion strain, albeit to a much lesser extent than the WT germlings (Fig 11B). This suggests that rescue by IBMX may simply be due to upregulation of redundant chitin deacetylases, as observed in *cbp1*, rather than the activation of an otherwise inactive signalling pathway.

Germinations were repeated on a hydrophilic glass surface (Fig 11C). No appressorium development was observed in the WT strain after a 24 hr incubation (consistent with previous observations [37]), but it was restored by the addition of IBMX or HDD. Both such inducers remained ineffective on *cda2/cbp1/cda3*. As exogenous chitosan fully restores appressorium development in the CDA deletion strains on a hydrophobic surface we extended this work to evaluate its effect on a hydrophilic surface. WT and *cda2/cbp1/cda3* conidia, germinated in the presence of 0.01% (w/v) chitosan for 24 hr did not form appressoria. Next, combined treatments of chitosan and IBMX or with HDD were applied. Interestingly, these combinatorial treatments induced appressorium development in the *cda2/cbp1/cda3* mutant. IBMX and HDD added in combination did not, however, induce appressorium development in the triple deletion strain. These experiments reveal two important facts: i) Chitosan acts independently of surface hydrophobicity, and ii) Chitosan relays through a separate pathway from IBMX or HDD.

### Chitosan is required for germling adhesion

**Fig 12:**
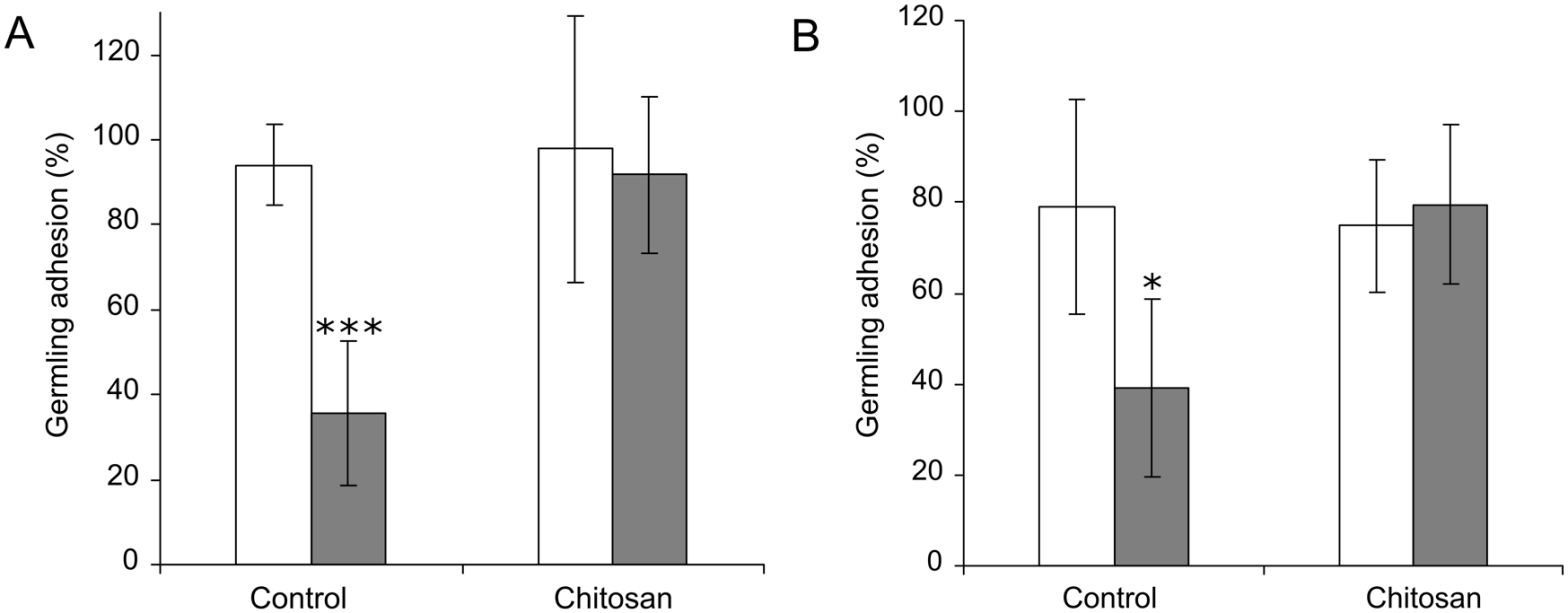
Germling adhesion is reduced in the *cda2/cbp1/cda3* strain. Adhesion of WT (white bars) and *cda2/cbp1/cda3* germlings (grey bars) on **A**) hydrophobic and **B**) hydrophilic glass surfaces with or without 0.01% chitosan (w/v). Conidial suspensions of equal concentrations were inoculated onto each surface and incubated for 2 hr, followed by 5 min washing with H_2_O, on an orbital shaker at 100 rpm. Germling adhesion was significantly reduced in the triple deletion strain (Student's T-test, *** p < 0.001, * p < 0.05), but restored by the addition of exogenous chitosan. Error bars: SD, n = 4.

During the course of these assays it was observed that *CDA* deletion strain germlings washed off artificial surfaces more readily than the WT strain. The percentage of WT and *cda2/cbp1/cda3* germlings adhering to hydrophilic and hydrophobic glass surfaces was quantified at 2 hpi, at which point appressorium development had not yet occurred. Germlings of the triple deletion strain demonstrated significantly lower adhesion than the WT, on both surfaces (Student's T-test, p < 0.05) (Fig 12A & 12B). This loss of adhesion could not be explained by lower conidial germination in the *cda2/cbp1/cda3* strain; 95% (±2 (SD), n = 3) of WT conidia had germinated by 2 hpi, compared with 98% (±1 (SD), n = 3) in *cda2/cbp1/cda3*. Remarkably, however, adhesion was fully restored on both surfaces by germinating the conidia in the presence of 0.01% chitosan (w/v), (Fig 12A & 12B). The adhesion of fungal cells to surfaces such as glass or plastic is mediated by cell wall glycoproteins [51]. These can be detected by staining with the fluorescently-labelled mannose-binding lectin Concanavalin A (FITC-ConA). Staining of WT or *cda2/cbp1/cda3* germlings at 2 hpi with FITC-ConA revealed that staining intensity was often reduced in *cda2/cbp1/cda3* strain (S11 Fig). Quantification of fluorescence intensity revealed significant (p < 0.01) differences between the WT and *cda2/cbp1/cda3* germlings in 3 of the 4 experiments (2-way ANOVA with post-hoc Tukey test) (S11 Fig).

## Discussion

Chitin deacetylases are a conserved family of enzymes in fungi. They catalyze the removal of acetyl groups from chitin, forming chitosan. It has been hypothesized that the deacetylation of chitin may either act as a 'stealth mechanism' to prevent the activation of a PAMP-triggered immune response in the host plant, or result in crucial alterations to the physical and chemical properties of the cell wall necessary for cellular development and morphogenesis. In this study, we present evidence to refute these hypotheses. Instead, we show that chitin deacetylation during germling morphogenesis is not an absolute requirement for either appressorium development or for pathogenicity. Instead, chitin deacetylation plays a role in perception of physical stimuli during the early stages of germling morphogenesis.

Appressorium development is induced by the perception of physical and chemical stimuli present on the plant surface, with signals then relayed via two key regulatory pathways in *M*. *oryzae*. The Pmk1 pathway operates at the earliest stage of appressorium development, and is required for sensing of wax, and possibly for surface attachment. The cAMP pathway, is hypothesized to operate at the commitment phase of appressorial development, and is required for sensing hydrophobicity and cutin monomers [49]. Considerable cross-talk exists between these two pathways, but activation of both is required for appressorium development. Many previous studies have been devoted to characterizing the proteins operating in these pathways (reviewed in [49]). Significantly, deletion of the genes encoding such proteins results in phenotypes that are similar to those reported for the *cda* mutants. The data from our study, set in the context of previous data on signal perception in *M*. *oryzae* [49] allows the role played by chitosan to be elucidated.

When germinated on an artificial hydrophobic surface, conidia of the *CDA* deletion strains *cbp1*, *cda2/cbp1* and *cda2/cbp1/cda3* failed to undergo early differentiation events, and appressorium development was either severely delayed (in *cbp1*) or rarely occurred at all (in *cda2/cbp1* and *cda2/cbp1/cda3*). Failure to differentiate appressoria on artificial surfaces is a phenotype shared by a number of other deletion strains with putative roles in surface sensing. For example, deletion strains of the signalling mucin Msb2, produce highly elongated, undifferentiated germ tubes on an artificial hydrophobic surface [48], but appressorium development is fully restored when inoculated onto a plant surface, just as in the *cda* strains described herein. In addition, cutin monomers were also unable to rescue *msb2*, although the effect of cAMP was not determined. Is there a functional link between Msb2 and chitosan? Msb2 has a single transmembrane domain, which anchors the protein to the plasma membrane, whilst the extracellular portion of the protein resides within the cell wall. This extracellular domain alone can partially suppress the defects associated with *MSB2* deletion [52]. Chitosan may be required for cell wall-localization of Msb2; the absence of chitosan may result in loss of Msb2 from the cell wall, resulting in the observed phenotype. However, the precise mechanism by which Msb2 mediates surface sensing remains elusive and this limits speculation on the putative functional link between this protein and chitosan. However, in a recent study, combined deletion of *CBP1* and *MSB2* had additive effects—virulence and Pmk1 phosphorylation (in vegetative hyphae) were shown to be more reduced in *msb2/cbp1* strains than in *msb2* strains [52]. Loss of chitosan may therefore affect multiple surface-sensing related processes at the earliest stage of germling morphogenesis, which converge on the Pmk1 pathway. In support of this hypothesis, *cbp1* germlings have also been shown to be unable to form hyphopodia on artificial hydrophilic surfaces, a phenotype shared by the *pmk1* mutant, but not the *cpka* mutant [34].

Other proteins with putative roles in surface sensing are the hydrophobin Mpg1 [43, 53], and the putative G-protein coupled receptor Pth11 [54]. The *mpg1* and *pth11* mutants demonstrate similar defects in appressorium development. That is, germlings produce elongate germ tubes on hydrophobic surfaces, and fail to develop appressoria, as in the *cda* mutants. There are, however, several important differences in the phenotypes: Firstly, appressorium development is also defective on plant surfaces in *mpg1* and *pth11*. Secondly, germ tubes of *mpg1* and *pth11* do undergo early differentiation events (germ tube hooking). Thirdly, cAMP can restore appressorium development in both *mpg1* and *pth11* [53, 54], but not in the *cda* mutants, although this is complicated by the presence of redundant CDAs that appear to be upregulated by cAMP [55, 56]. This suggests that Mpg1 and Pth11 act upstream of the cAMP pathway, whereas chitosan may not. This hypothesis is supported by the fact that chitosan and IBMX/cutin had synergistic effects on appressorium development on a hydrophilic surface. Yet, the fact that germination in the presence of 1-octacosanol, which acts upstream of the Pmk1 pathway (perceived by the ShoI receptor protein) [48], could only partially restore appressorium development in the *cda2/cbp1/cda3* strain, may suggest that activation of the cAMP pathway is also defective in this mutant. On the other hand, cutin monomers, which act through the cAMP pathway [54], did not have a synergistic effect with wax, which may suggest the cAMP pathway is already active in the *cda2/cbp1/cda3* strain. Partial rescue with 1-octacosanol could instead be explained by the composition of the wax, which may not effectively mimic the mixture present on leaf surfaces [57].

An alternative explanation for the observed phenotypes in the *cda* mutants, although one that is not mutually exclusive with those proposed above, is that the lack of germling adhesion is responsible. Germlings of *cda2/cbp1/cda3* were much more easily detached than those of the WT, even under relatively gentle washing conditions. This suggests that the adhesive properties of the germ tube are defective in the absence of chitosan. In this scenario, germ-tube localized chitosan is required for the adhesion of the germ tube to the surface itself, and is therefore required for surface sensing, since such sensing mechanisms presumably require close contact between the germ tube and the surface in question. Thus, chitosan may act directly as a polysaccharide adhesin, analagous to the role of galactosaminogalactan (GAG) in the cell wall of *Aspergillus fumigatus*, which mediates adhesion to hydrophobic surfaces and host cells [58, 59]. Alternatively, chitosan may be required for the attachment of various adhesin-like proteins to the cell wall (or a combination of both). Lack of chitosan in *cda2/cbp1/cda3* did result in a reduction of Concanavalin A staining, suggesting a reduction of mannan in the cell wall, but there are several possible explanations for this: i) Chitosan is directly required for attachment of mannoproteins to the cell wall, ii) Loss of chitosan results in large-scale changes in cell wall composition, affecting attachment of mannoproteins or resulting in a reduction in non-protein associated mannan, iii) Mannoproteins are produced, but secretion to the outer wall is affected by loss of chitosan or iv) Lack of surface sensing and activation of Pmk1 and/or cAMP pathways means that production of the mannoproteins does not occur in the first place, i.e. reduced germling adhesion is a consequence, and not the cause of defective surface sensing. Further investigations are required to distinguish between these possibilities.

The rescue of appressorium development in the *cda* mutants by exogenous chitosan and its association with the cell wall was an unexpected result, and it is unclear exactly how this occurs. Because the charge of the exogenous chitosan was of great importance, this may indicate that an electrostatic interaction is responsible for its association with the cell wall. This, in turn, suggests the existence of an anionic cell wall component, which may form a complex with the cationic chitosan. At present, little is known of anionic cell wall components in fungi. Uronic acids are known to be present in the cell walls of some fungi [60–63], as is phosphomannan [64–66], but their presence in *M*.*oryzae* has not been investigated. In addition to an electrostatic association, the possibility of enzymatic incorporation of exogenous chitosan must also be considered. CRH transglycosylases have previously been shown to be able to incorporate exogenous fluorescently-labelled oligosaccharides in *S*.*cerevisiae* [67]. It is therefore possible that other enzymes with similar activities may be responsible for the hypothesized incorporation of exogenous chitosan.

**Fig 13:**
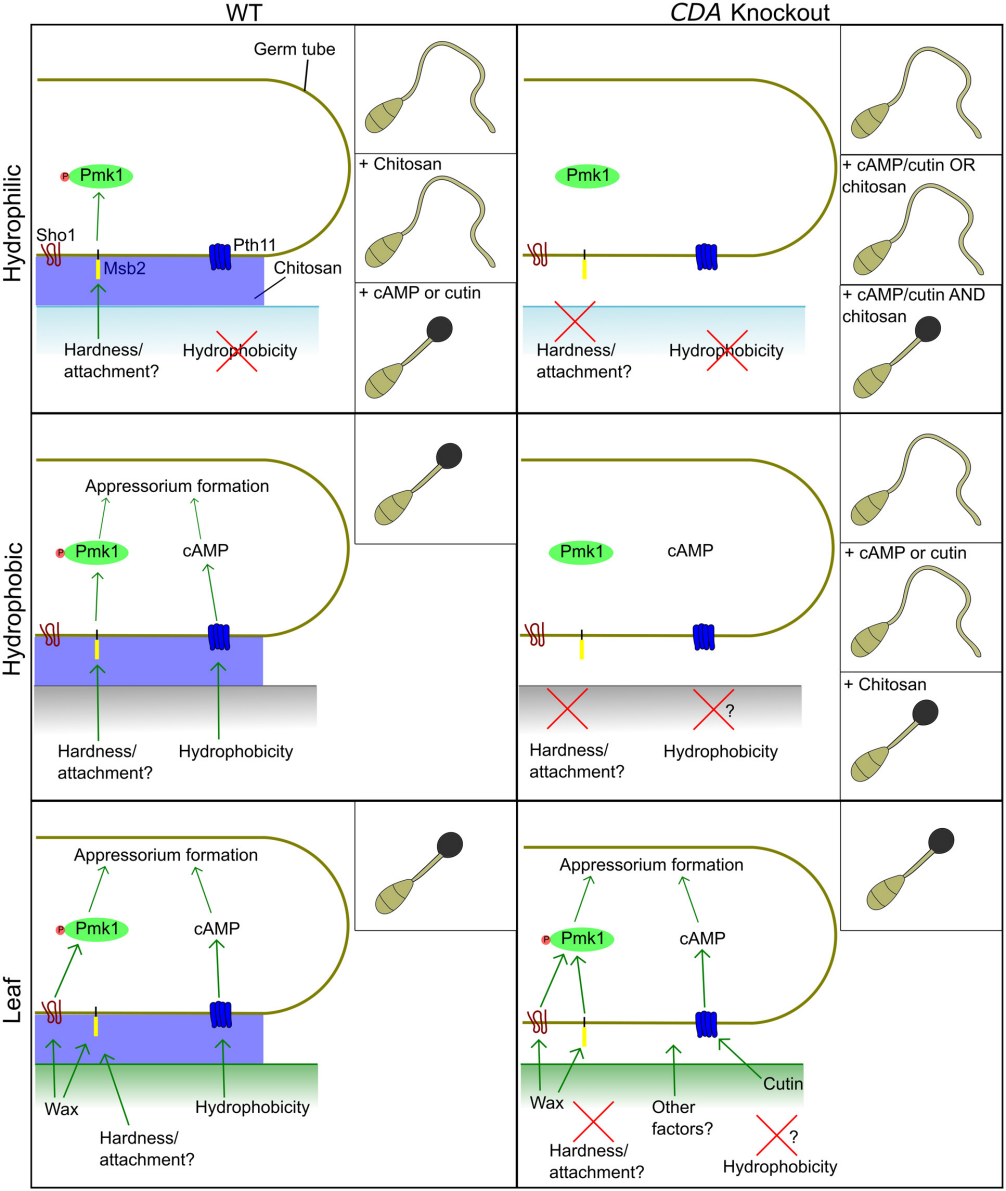
Model describing the role of chitosan in appressorium development. Germling development on three different surfaces is shown (hydrophilic, hydrophobic and leaf), with observed phenotypes shown inset. Successful appressorium development is dependant upon activation of both the Pmk1 and cAMP pathways. The absence of chitosan prevents complete activation of the Pmk1 pathway, and possibly the cAMP pathway. The addition of chitosan and/or chemical inducers of these pathways restores appressorium development in the *CDA* deletion strains. Thus, germ tube localized chitosan likely acts upstream of both the Pmk1 and cAMP pathways to induce appressorium development.

The role of chitosan in appressorium development is an intriguing one, and there remain several unresolved issues, as discussed above. Despite this, the model presented in Fig 13 is consistent with all of the data presented herein, and with previous studies characterizing surface sensing proteins in *M*.*oryzae*. In this model, germ-tube localized chitosan is required for the activation of the Pmk1 MAP kinase pathway in response to a surface, but independently of surface hydrophobicity. Optimal activation of the cAMP pathway may also require chitosan, but it is not possible to conclusively determine whether just one or both of the signalling pathways are defective in *cda* mutants from the current data.

The deacetylation of chitin was hypothesized to cause profound changes to the chemical and physical properties of the cell wall, which could be crucial for cellular morphogenesis. However, no evidence for this has been found in the present study. Germlings of the *cda2/cbp1/cda3* strain developed appressoria that were morphologically indistinguishable from the WT strain on plant surfaces, despite the absence of detectable chitosan. Additionally, appressorium development in *cda2/cbp1/cda3* was restored by a short incubation with FITC-chitosan that was only incorporated into the germ tube. Not only was chitosan not required for morphogenesis of appressoria, but appressorium function also seemed to be unaffected by the absence of chitosan, since plant penetration continued to be achieved successfully. Chitosan is therefore also unlikely to be a key structural component of the cell wall, in contrast to *Cryptococcus neoformans* where loss of chitosan impaired cellular integrity [31]. On the other hand, it is also possible that compensatory changes in cell wall composition occurred in the absence of chitosan, which were not investigated. The upregulation of other cell wall components may be sufficient to maintain cell wall integrity in the *cda* mutants. In a similar vein, it is not inconceivable that compensatory changes were also responsible for allowing appressorium morphogenesis to occur in the absence of chitosan i.e. there are multiple, redundant morphogenic mechanisms operating during appressorium development.

A second hypothesized role of chitosan is in the protection from plant chitinases. However, the findings presented here, together with evidence from previous studies do not support this role for chitosan, at least in germlings. Germlings of *cda2/cbp1/cda3* in which chitosan was absent remained intact on leaf surfaces, in contrast to those of the *ags* mutant which lack α1,3-glucan and were destroyed, presumably by degradative enzymes on the leaf surface [18]. This suggests that α1,3-glucan may be the cell wall component with the protective role in *M*.*oryzae* germlings. It would also be valuable to investigate the hypothesized protective effects of chitin deacetylation during *in planta* growth. Chitosan staining was not performed on invasive hyphae in the *cda* mutants, although qRT-PCR analysis suggested that the chitin deacetylases operating during appressorium development are not highly expressed during invasive growth (S2 Fig). Instead, *CDA1* and *CDA6* appear to be involved in chitin deacetylation at this stage of infection. Further investigations should therefore focus on the characterization of these genes to determine the role of chitosan in invasive hyphae.

The deacetylation of chitin is hypothesized to require a degree of collaboration between the membrane-localized chitin synthases, and cell wall or membrane-associated chitin deacetylases [31, 68]. Evidence from this study may lend further support to this hypothesis. Deletion of *CHS7* results in an almost identical phenotype to multiple *CDA* deletion [69], suggesting that Cbp1, Cda2 and Cda3 may deacetylate the chitin synthesized by Chs7. Further investigation is required to determine the relationship between chitin synthesis and deacetylation during appressorium development in *M*.*oryzae*.

In summary, the investigation of chitin deacetylation during appressorium development in *M*.*oryzae* has yielded unexpected and intriguing data. The deacetylation of chitin by at least three chitin deacetylases, with overlapping roles, is required for surface sensing in germlings. Yet, this requirement is surface dependant, due to the multiple, redundant mechanisms by which appressorium development can be induced in *M*.*oryzae*. Nevertheless, this study provides a novel insight into the mechanisms behind the perception of physical stimuli in *M*.*oryzae*, and also demonstrates a novel way in which the cell wall is crucial in acting as an interface between fungal cells and their environment. Importantly, evidence in support of the long-standing hypothesis regarding the role of chitin deacetylation was not found; chitosan is not required as a 'stealth' mechanism in germlings of *M*.*oryzae*, and so the role of this cell wall component in the interactions between phytopathogenic fungi and their hosts may need to be reconsidered. However, further work is required to determine whether or not chitosan acts as a 'stealth mechanism' during *in planta* growth of *M*.*oryzae*, as this was not determined in this study.

## Materials and Methods

### Fungal strains and growth conditions

The wild-type (WT) rice pathogenic *Magnaporthe oryzae* strain Guy11 and mutant strains were cultured at 24°C with a 14 h light 10 h dark cycle. Strain maintenance and composition of media were essentially as described by Talbot *et al* [43].

### RT-PCR

RNA was extracted from rice leaves inoculated with conidia of the Guy11 strain using the Qiagen RNeasy RNA extraction kit, according to manufacturer's instructions. RNA concentration was determine using a ThermoScientific ND-1000 NanoDrop spectrophotometer, integrity was evaluated by gel electrophoresis. Genomic DNA was removed by using an RNase free DNase set (Qiagen), according to manufacturers’ instructions. Reverse transcription of 1 μg of RNA into cDNA was performed by using the Maxima First Strand cDNA synthesis kit (ThermoFisher Scientific), according to manufacturers instructions, using random hexamer primers. qRT-PCR was performed with Power SYBR Green PCR master mix (ThermoFisher Scientific), on an Applied Biosystems 7300 Real-time PCR system. Primers are listed in S2 Table (37–58). Primers were designed to span an intron where possible (not all *CDA* genes have introns), and efficiency of the primers was 85–104% (average efficiency 93.6%). No amplification was observed in samples that were not reverse transcribed (-RT control), in samples without RNA (NTC control), or in RNA extracted from mock-inoculated leaves. Relative transcript abundance was calculated by the efficiency correction method [70] as follows: Abundance = E_target_^(Ct(reference)-Ct(target))^.

### Targeted deletion of *M*. *oryzae CDAs*

Single *CDA* deletion strains were generated by replacing the coding sequences of *CBP1* (*MGG_12939*), *CDA2* (*MGG_09159*) and *CDA3* (*MGG_04172*) with a hygromycin resistance cassette [71]. Briefly, sequences flanking the target genes (~1.5kb upstream, and ~1.1kb downstream) were amplified by PCR, using primers 7–10 (for *CBP1*), 13–16 (for *CDA2*) and 17–20 (For *CDA3*) (primers are listed in S2 Table). These fragments were joined to the hygromycin resistance gene by overlapping PCR, using primer pairs 7/4 and 3/10 (for the *CBP1* deletion construct), 13/4 and 3/16 (for the *CDA2* deletion construct), 17/4 and 3/20 (for the *CDA3* deletion construct). The final, complete construct was made by overlapping PCR, amplifying the products from the previous reactions using primer pairs 7/10 (for *CBP1)*, 13/16 (for *CDA2*) and 17/20 (for *CDA3*). The final PCR product was used directly for DNA-mediated protoplast transformation of WT Guy11 strain following protocols described by Talbot *et al* (39). Putative transformants were selected on minimal medium (MM) supplemented with 300 μg ml^-1^ hygromycin B (Calbiochem, Merck, Darmstadt, Germany). Deletion of the target gene was confirmed by both PCR and Southern Blot analysis, as described in Samalova *et al* [72].

Double *CDA* deletion strains were generated in the *cda2* or *cbp1* background strains. The coding sequences of *CBP1* and *CDA3* were replaced with a bialaphos resistance cassette. The deletion construct was made as described above, except primers 8 & 9 were substituted for 11 & 12 (for *CBP1*), and primers 18 & 19 for 21 & 22 (for *CDA3*). The *CBP1* deletion cassette was transformed directly into protoplasts of the *cda2* strain, to generate the *cda2/cbp1* mutant. The *CDA3* deletion cassette was transformed into both the *cda2* and *cbp1* background strains, to generate the *cda2/cda3* and *cbp1/cda3* mutants. Putative transformants were selected on defined complex medium (DCM) supplemented with 60 μg ml^-1^ Bialophos (Goldbio, St Louis, MO, USA). Deletion strains were confirmed as above.

To generate the triple *cda2/cbp1/cda3* mutant, *CDA3* was deleted in the *cda2/cbp1* mutant. In this case, the coding sequence of *CDA3* was replaced by a sulphonylurea resistant allele of the *M*.*oryzae ILV* gene (MGG_06868). Since the sulphonylurea transformation constructs were too large to be generated by over-lapping PCR, GAP-repair *S*. *cerevisiae* cloning [73] was used to assemble the constructs in pNEB1284 vector. Primers 5,6 and 23–26 were used to amplify the required DNA fragments. Putative transformants were selected on BDCM medium supplemented with 100 μg ml^-1^ chlorimuron ethyl (Sigma Aldrich, UK) and confirmed as specified above.

### Cloning of fluorescently tagged *CDAs*

Standard molecular techniques [74] were used to prepare the complementation constructs with fluorescently tagged *CDAs*. A set of transformation vectors based on pUCAP was generated as described in Samalova *et al*. [75]. The vectors contain polyadenylation signal pATrpC and either bialophos or hygromycin resistance marker that was cloned into re-created *Sal*I sites using primer pairs 1/2 or 3/4 respectively (see S2 Table and S12 Fig). For PCR amplification of *CBP1* and *CDA2*, primer pairs 27/28 and 29/30 were used, respectively. Genomic DNA from the WT strain Guy11 was used as a template, and amplified using Herculase DNA polymerase (Agilent). This resulted in amplification of the coding sequence of the genes (without stop codons), together with 2 kb of native promoter sequence for *CBP1* and 1.3 kb for *CDA2*. The PCR products were cloned into the *Asc*I sites of the vector described above (S12 Fig), creating C-terminal mCherry fusions.

### Confocal imaging

Conidia (2.5 x 10^5^ ml ^-1^) of Guy11 and complemented strains were collected from 10 day old plates and inoculated in 50 μl droplets onto hydrophobic glass cover-slip,; onion peels, or rice leaf sheaths; as described in Samalova *et*. *al*., [75] and incubated for specified times in the growth chamber. For viewing mCherry fluorescence, the samples were viewed using the C-Apochromat 40x/1.2 water corrected objective lens of a Zeiss LSM 510 Meta confocal microscope at 543 nm excitation from the HeNe laser and emission collected with BP565-615 filter for mCherry.

Calcofluor White staining was performed as follows: Conidia (2.5 x 10^5^ ml ^-1^) of the Guy11 and mutant strains were inoculated in 50 μl droplets onto hydrophobic glass cover-slips, and incubated for specified times in the growth chamber. After the incubation, the water droplet was removed from the cover-slip, and replaced with 100 μl of 0.05% Calcofluor White solution (Sigma-Aldrich, UK), and incubated for 20 min. Samples were then washed briefly with dH_2_O, and viewed using the Zeiss LSM510 microscope as above, with 405 nm excitation and emission collected with an LP420 filter.

OGA488 staining was performed essentially as described in [36]. Briefly, Conidia (2.5 x 10^5^ ml^-1^) of the Guy11 and mutant strains were inoculated in 50 μl droplets onto hydrophobic glass cover-slips, onion epidermis, or rice leaf sheaths and incubated for specified times in the growth chamber. Samples were washed briefly with 25 mM MES (pH 5.6), and incubated with OGA488 (a generous gift from William Willats [36]) (diluted 1/1000 in 25 mM MES) for 15 min on ice. This was followed by 2–3 brief washes with 25 mM MES, after which the samples were viewed using the C-Apochromat 40x/1.2 water corrected objective lens of a Zeiss LSM 510 Meta confocal microscope, at 488 nm excitation and emission collected with an LP505 filter.

Staining of germlings with FITC-labelled Concanavalin A (ConA-FITC) (Sigma-Aldrich, UK) was performed as follows: Conidia (2.5 x 10^5^ ml^-1^) of the Guy11 and mutant strains were inoculated in 50 μl droplets onto hydrophobic glass cover-slips, and incubated in the growth chamber for 2 hr. Cover-slips were washed briefly with PBS, then incubated with 40 μg/ml ConA-FITC for 20 min on ice, then washed briefly with PBS 2–3 times. Samples were viewed using the C-Apochromat 40x/1.2 water corrected objective lens of a Zeiss LSM 510 Meta confocal microscope, at 488 nm excitation and emission collected with an LP505 filter. Fluorescence intensity was quantified using ImageJ.

Staining with the monoclonal anti-chitosan antibody mAbG7 (a generous gift from Stefan Schillberg [35]) was performed as follows. Conidia (2.5 x 10^5^ ml^-1^) of the Guy11 strain were inoculated in 50 μl droplets onto hydrophobic glass cover-slips, and incubated in the growth chamber for the specified time. Samples were first blocked by incubation with 2% BSA (w/v) in PBS for 1 hr at room temperature. Samples were washed 3 times with PBS/T (PBS +0.05% Tween 20), for 5 min each on an orbital shaker (~70 rpm) and then incubated with the primary antibody (mAbG7, at 10 μg/ml in PBS) for 1.5 hr at room temperature. This incubation was followed by 3 more washing steps as described above, and incubation with the secondary antibody (FITC-labelled anti Mouse IgM, 5 μg/ml in PBS) at room temperature for a further 1.5 hr. Finally, the secondary antibody was removed by 3 more washing steps with PBS/T as described previously and viewed under using the Zeiss LSM510 microscope, as described above for ConA-FITC staining. A negative control was included in all experiments, in which samples were only incubated with the secondary antibody.

Staining with the polyclonal anti-chitosan antibody (a generous gift from Holger Deising [19]) was performed as for the mAbG7, except that the antibody was used at a dilution of 1/100 from the original antiserum, and the secondary antibody (a FITC-labelled anti-rabbit IgG) was used at 10 μg/ml (in PBS).

### Pathogenicity and infection-related morphogenesis assays

Conidial germination and appressorium development were assessed at 1, 8, 16 or 24 hpi by following germling differentiation on hydrophobic glass cover-slips (Gerhard Menzel, Glasbearbeitungswerk GmbH & Co., Braunschweig, Germany). Conidia (2.5 x 10^5^ ml^-1^) of the Guy11 and mutant strains were inoculated in 50 μl droplets onto hydrophobic glass cover-slips, and incubated in the growth chamber for the specified time. Samples were viewed under an Olympus BX50 microscope, and ~500 germlings in 3 independent experiments counted per strain/timepoint. For germinations in the presence of chemical inducers of appressorium development, IBMX (Sigma-Aldrich, UK) was used at 2.5 mM (from a 250 mM stock in DMSO), 1,16 hexadecanediol (Sigma-Aldrich, UK) at 200 μM (from a 50 mM stock in ethanol) and diacylglycerol (1,2-dioctanoyl-*sn*-glycerol) (Enzo Life Sciences) at 58 μM (from a 7.25 mM stock in DMSO). Chitosan and its derivatives were used at a final concentration of 0.01% or 0.001%, from a 1% stock (except for FITC-chitosan which was from a 0.08% stock). For germinations in the presence of wax (1-octacosanol) (Sigma-Aldrich, UK), the wax was first dissolved in chloroform to a concentration of 4 mg/ml. In a laboratory fume hood, 100 μl of this stock was pipetted onto a hydrophobic glass coverslip and the chloroform allowed to evaporate, leaving a layer of wax on the coverslip. Conidia were then inoculated onto this surface, as described above. For germinations on hydrophilic glass coverslips (Heathrow Scientific) the protocol was identical except that only 20 μl of conidial suspension was used. Coverslips were placed in square Petri dishes with damp filter paper and sealed with Parafilm to prevent evaporation.

Cuticle penetration was assessed by scoring the frequency with which appressoria formed penetration pegs and intracellular infection hyphae on rice leaf sheaths, after incubation at 24°C for 24 h.

Leaf infection assays were performed on blast-susceptible, 21-day-old seedlings of rice (*Oryza sativa* L.) cultivar CO39. Assays on detached leaves were performed as described in [72]. For assays on whole plants, 21-day-old seedlings of rice (*Oryza sativa* L.) cultivar CO39 were spray inoculated with 4 ml of conidial suspension at three different concentrations (1.25 x 10^5^, 6.25 x 10^4^ or 3.13 x 10^4^ conidia/ml, in 0.2% (w/v) gelatine water). A mock inoculation of 0.2% (w/v) gelatine water was included as a negative control. Infection was assessed 4 days later.

### Germling adhesion assay

Adhesion assays were performed on both hydrophobic and hydrophilic glass coverslips. 20 μl droplets of conidial suspension (either with or without 0.01% chitosan) of each strain were pipetted onto the coverslips, which were placed into a humidity chamber and incubated at 24°C for 2 hr to allow germination. At 2 hpi, half of the coverslips were removed and placed into a 50 mm Petri dish containing 5 ml dH2O, and shaken on an orbital shaker at 100 rpm for 5 min. Coverslips were then removed and mounted on slides for viewing under the 10X objective lens of an Olympus BX50 microscope. Conidia were counted from one field of view (for hydrophobic coverslips) or three fields of view (for hydrophilic coverslips), with a conscious effort made to locate the area of the coverslip with the highest conidial density. For each strain, the number of conidia counted on the washed coverslips was compared with those counted on the unwashed coverslips, and percentage conidial adhesion calculated as: 100—((conidia on unwashed coverslip—conidia on washed coverslip) /conidia on unwashed coverslip) x 100.

### Synthesis of FITC-chitosan

FITC-labelled chitosan was synthesized exactly as described previously [45].

## Supporting Information

S1 Fig Alignment of *M*. *oryzae* CDA protein sequences with those from *C*. *lindemuthianum* and *S*. *cerevisiae*, using Clustal Omega. Conserved active site residues are marked in green boxes, and the zinc binding triad in blue boxes.
(TIF)

S2 Fig Expression of chitin deacetylases during appressorium development. **A**) Development of appressoria (black arrow head) at 5 hpi on rice leaves. **B**) Melanized appressoria (white arrow head) and invasive hyphae (black arrow head) at 36 hpi on rice leaves. Scale bars: 20 μm. **C**) Expression of *CDA*s during *in planta* development at 36 hpi. Error bars show standard deviation, n = 3.
(TIF)

S3 Fig PCR analysis of *CDA* deletion strains. **A**) Schematic of deletion strategy, whereby the gene of interest is replaced by a gene encoding antibiotic resistance. Arrows show position of primers used to screen transformants. Pair 1 amplifies the gene of interest, confirming its absence in the deletion strain. Pairs 2 and 3 test for integration of the deletion construct at the desired locus. **B**) PCR analysis of single *CDA* deletion strains, showing successful deletion of *CBP1*, *CDA2* and *CDA3*. **C**) PCR analysis of double *CDA* deletion strains (gene tested for written in red). **D**) PCR analysis of triple *CDA* deletion strain, showing successful deletion of *CDA3*. Note: Some of the gel pictures are composites images of different parts of the same gel.
(TIF)

S4 Fig Southern Blot analysis of *CDA* deletion strains. **A**) Targeted gene deletion strategy, whereby the gene of interest is replaced by a gene encoding resistance to an antibiotic, due to homologous recombination between sequences (green). Southern blots showing successful single **B**), double **C**) and triple **D**) knockouts of chitin deacetylases.
Blots containing restriction digested gDNA of putative deletion strains were hybridised with α-^32^P labelled DNA homologous to the hygromycin, bialaphos or sulphonylurea resistance genes respectively. The cartoon above each blot shows the expected band size based upon the position of the restriction enzyme sites at each locus. Successful gene replacement is evidenced by a single band of the expected size, (as calibrated against standard gel size marker ladder and with band sizes given in kilobases (kb). For the double deletion strains, cross-hybridisation was observed between the hygromycin and bialaphos resistance cassettes, due to a common promoter sequence. These faint bands are labelled in **B**.
(TIF)

S5 Fig Germination of WT and *cda2/cbp1/cda3* conidia on plastic and parafilm. Conidia of the WT and *cda2/cbp1/cda3* strains were inoculated onto the surfaces in 50 μl droplets, and incubated for 24 hr. Scale bars: 40 μm.
(TIF)

S6 Fig Staining of WT and *cda* deletion strains with OGA488 or Calcofluor White. **A**) OGA488 staining of WT, *cda2/cbp1* and *cda2/cbp1/cda3* germlings at 24 hpi, showing absence of labelling in appressoria of the deletion strains (white arrow heads). **B**) Calcofluor white staining of germlings at 16 hpi, showing abnormally elongated, septate germ tubes (white arrow heads) in mutant strains, and abnormally shaped appressoria in *cbp1*. Scale bars: 20 μm.
(TIF)

S7 Fig **A**) Cbp1:mCherry and **B**) Cda2:mCherry fluorescence at later stages of appressorium development (as indicated). Scale bars: 10 μm.
(TIF)

S8 Fig Formulations and derivatives of chitosan tested for their ability to rescue appressorium development. Molecular structure, degree of polymerization and degree of deacetylation (DDA) are indicated (when known).
(TIF)

S9 Fig Pathogenicity of the *cda* mutants on detached rice leaves. A) Pathogenicity of the *cda2/cbp1/cda3* deletion strain on rice leaves, showing comparable lesion density to the WT strain (two independent triple deletion lines are shown). A mock inoculation of 0.2% gelatine was included as a negative control. Scale bars: 1 cm. **B**) Quantification of lesion numbers on detached rice leaves inoculated with different *cda* mutant strains, normalized to the Guy11 WT strain (± SD, n = 3).
(TIF)

S10 Fig Germlings of the WT or *cda2/cbp1/cda3* strain stained with Calcofluor White. Appressorium development was observed in *cda2/cbp1/cda3* (white arrow heads) in the presence of 1-octacosanol, but germ tubes remained abnormally elongated. Scale bars: 50 μm.
(TIF)

S11 Fig Concanavalin A staining of WT and *cda2/cbp1/cda3* germlings. **A**) Germlings of the WT and *cda2/cbp1/cda3* strain, stained with FITC-ConA. Pictures were taken from Experiment 1 (quantified in B). Scale bars: 50μm. **B**) Fluorescence intensity of germlings stained with FITC-ConA. Fluorescence intensities of ~140 germ tubes were measured, across 4 independent experiments. White bars: WT, grey bars: *cda2/cbp1/cda3*. Significant differences were found in 3 of the 4 experiments (2-way ANOVA with post-hoc Tukey test (** = p <0.01, *** = p < 0.001))
(TIF)

S12 Fig Map of the pUCAP vector used for cloning of fluorescent gene fusions. (TIF)

S1 Table List of strains used in this study. (XLS)

S2 Table List of primers used in this study. (XLS)

S3 Table Germ tube lengths (in μm) of WT and *cda* mutant germlings. Conidia of the WT and *cda* mutants were inoculated onto an artificial surface and incubated for 16 hr, in the presence of IBMX (3-isobutyl-1-methylxanthine), HDD (1,16 hexadecanediol; cutin monomer) or DAG (1,2-dioctanoyl-*sn*-glycerol). Significant (Mann-Whitney U-test, p < 0.001) reductions (asterisks) in germ tube lengths were observed in the deletion strains with all treatments, when compared with the control. ND—Not determined.
(TIF)
