# Supplementary figures and images for: Chitosan Mediates Germling Adhesion in Magnaporthe oryzae and Is Required for Surface Sensing and Germling Morphogenesis
Source: PLoS Pathog. 2016 Jun 17;12(6):e1005703. doi: 10.1371/journal.ppat.1005703 (PMC4912089; doi:10.1371/journal.ppat.1005703)

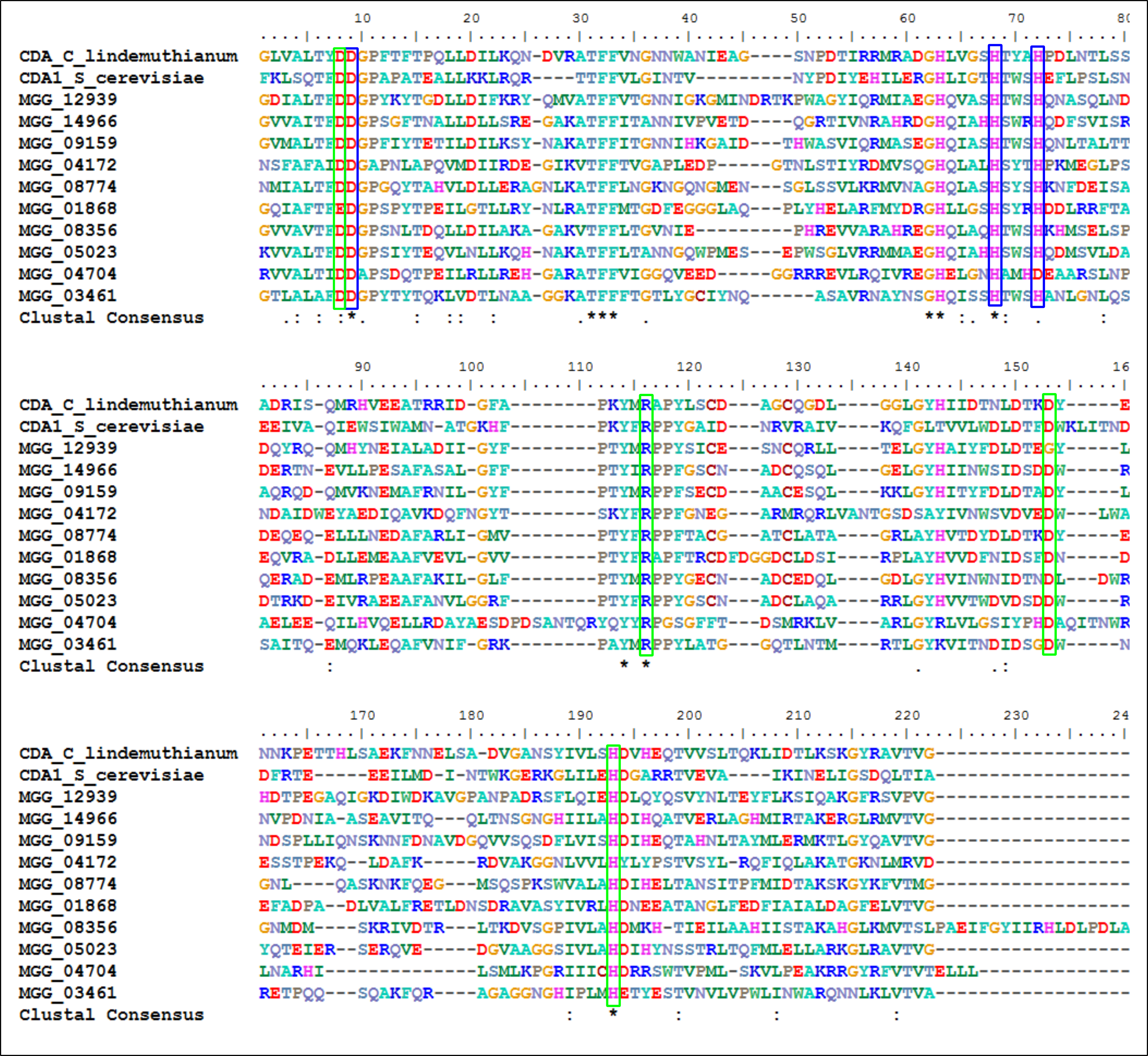

Supplement: S1 Fig — Conserved active site residues are marked in green boxes, and the zinc binding triad in blue boxes. (TIF) [file ppat.1005703.s001.tif]

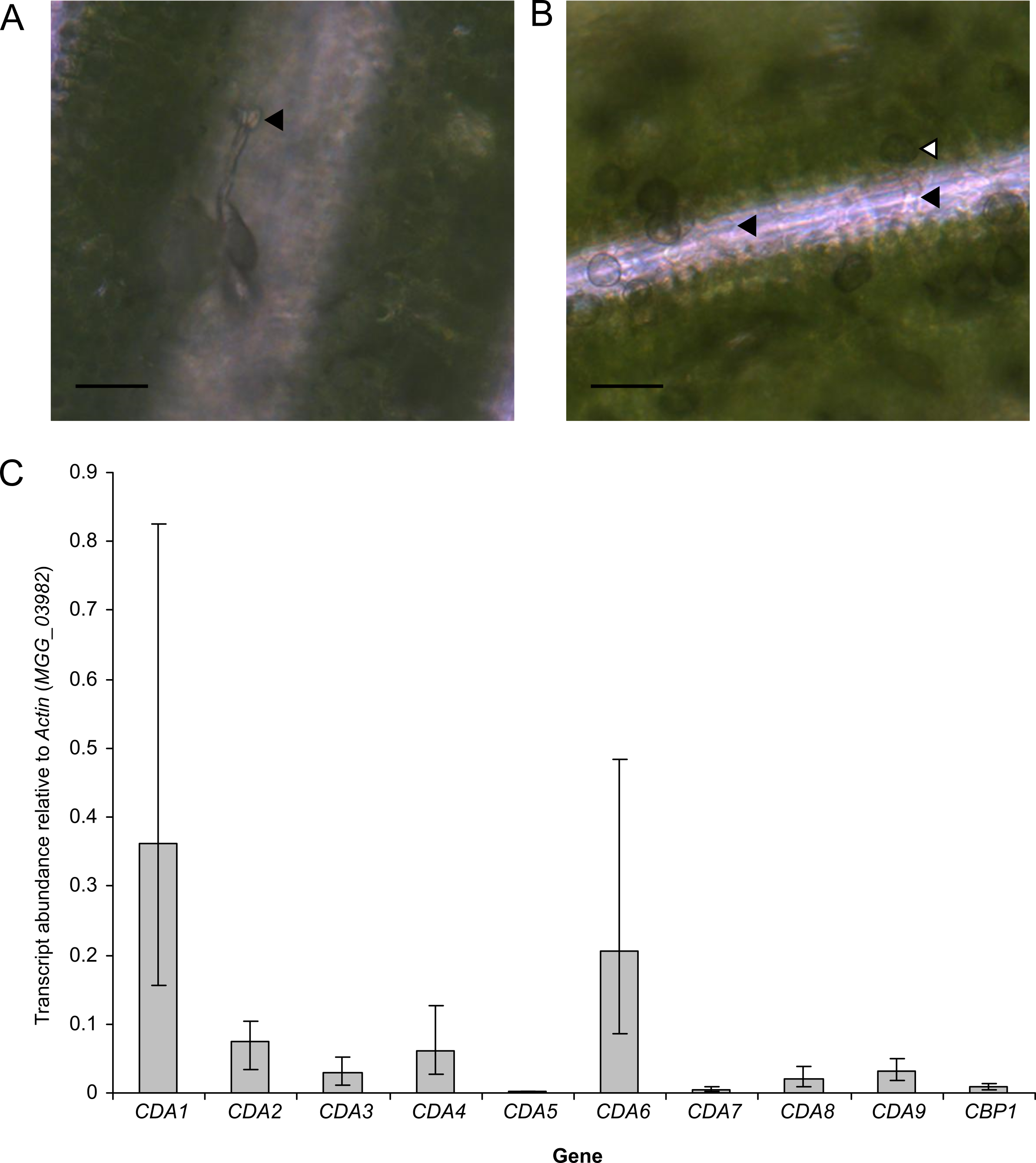

Supplement: S2 Fig — A) Development of appressoria (black arrow head) at 5 hpi on rice leaves. B) Melanized appressoria (white arrow head) and invasive hyphae (black arrow head) at 36 hpi on rice leaves. Scale bars: 20 μm. C) Expression of CDAs during in planta development at 36 hpi. Error bars show standard deviation, n = 3. (TIF) [file ppat.1005703.s002.tif]

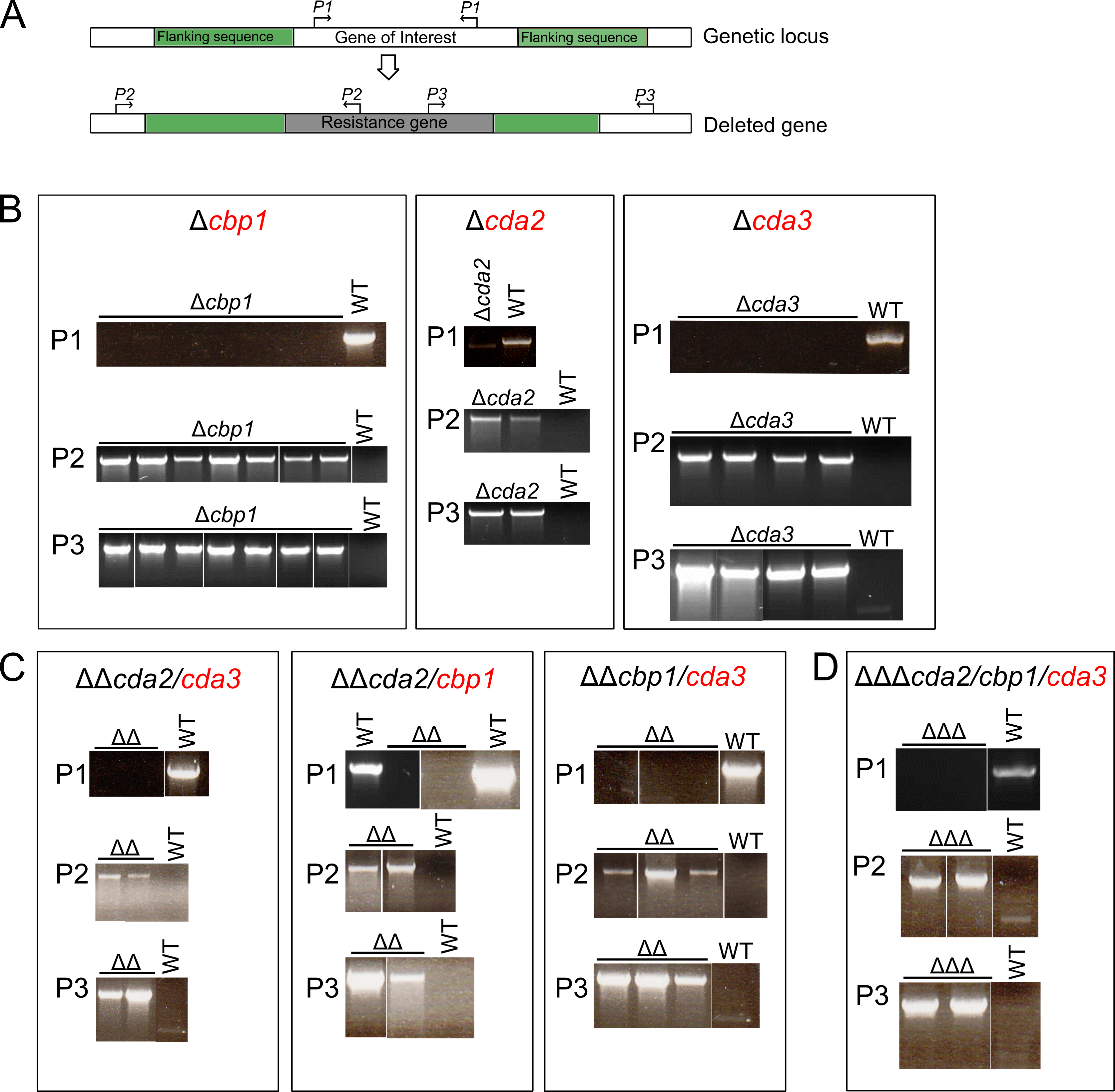

Supplement: S3 Fig — A) Schematic of deletion strategy, whereby the gene of interest is replaced by a gene encoding antibiotic resistance. Arrows show position of primers used to screen transformants. Pair 1 amplifies the gene of interest, confirming its absence in the deletion strain. Pairs 2 and 3 test for integration of the deletion construct at the desired locus. B) PCR analysis of single CDA deletion strains, showing successful deletion of CBP1, CDA2 and CDA3. C) PCR analysis of double CDA deletion strains (gene tested for written in red). D) PCR analysis of triple CDA deletion strain, showing successful deletion of CDA3. Note: Some of the gel pictures are composites images of different parts of the same gel. (TIF) [file ppat.1005703.s003.tif]

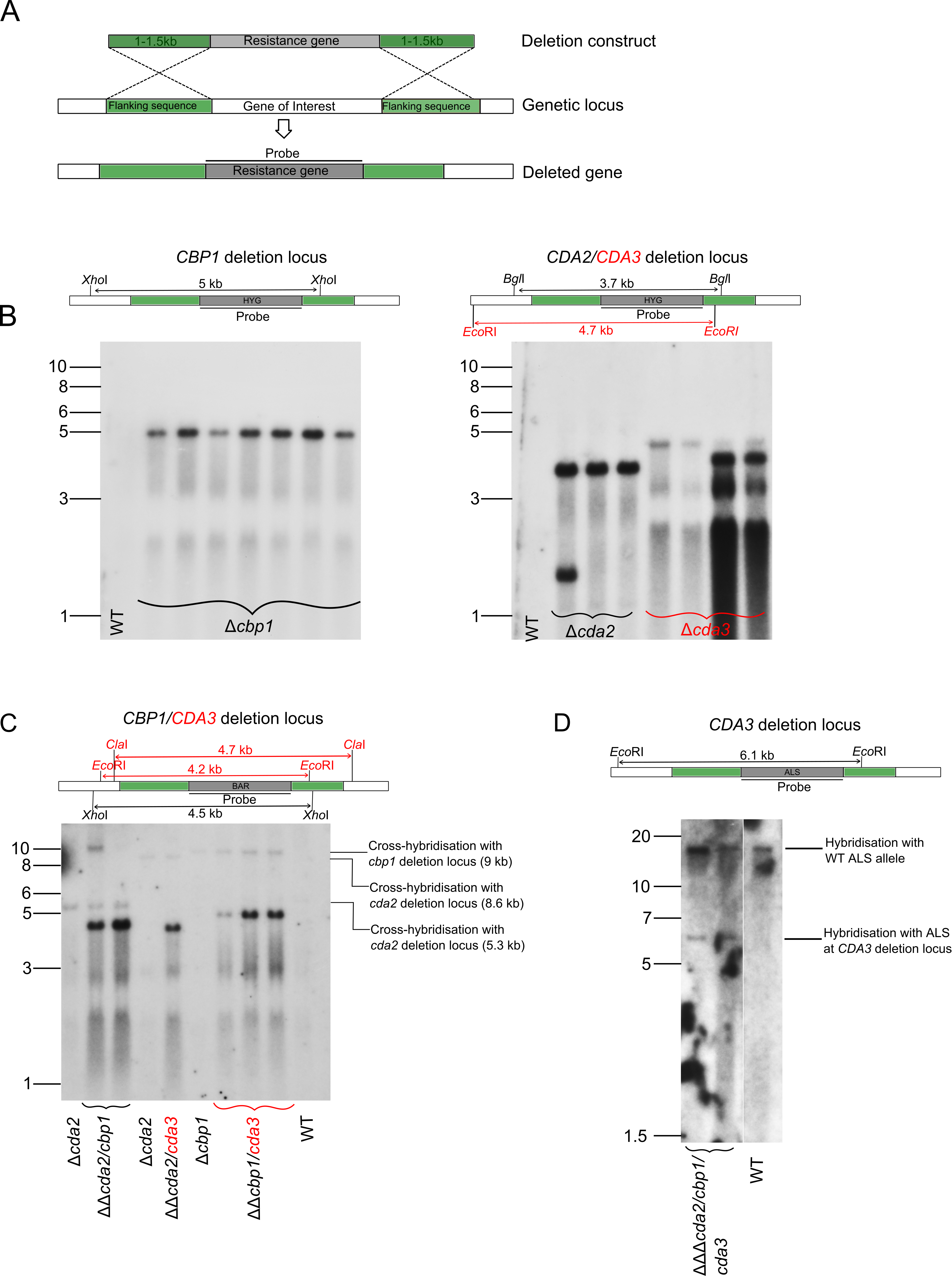

Supplement: S4 Fig — A) Targeted gene deletion strategy, whereby the gene of interest is replaced by a gene encoding resistance to an antibiotic, due to homologous recombination between sequences (green). Southern blots showing successful single B), double C) and triple D) knockouts of chitin deacetylases. Blots containing restriction digested gDNA of putative deletion strains were hybridised with α-32P labelled DNA homologous to the hygromycin, bialaphos or sulphonylurea resistance genes respectively. The cartoon above each blot shows the expected band size based upon the position of the restriction enzyme sites at each locus. Successful gene replacement is evidenced by a single band of the expected size, (as calibrated against standard gel size marker ladder and with band sizes given in kilobases (kb). For the double deletion strains, cross-hybridisation was observed between the hygromycin and bialaphos resistance cassettes, due to a common promoter sequence. These faint bands are labelled in B. (TIF) [file ppat.1005703.s004.tif]

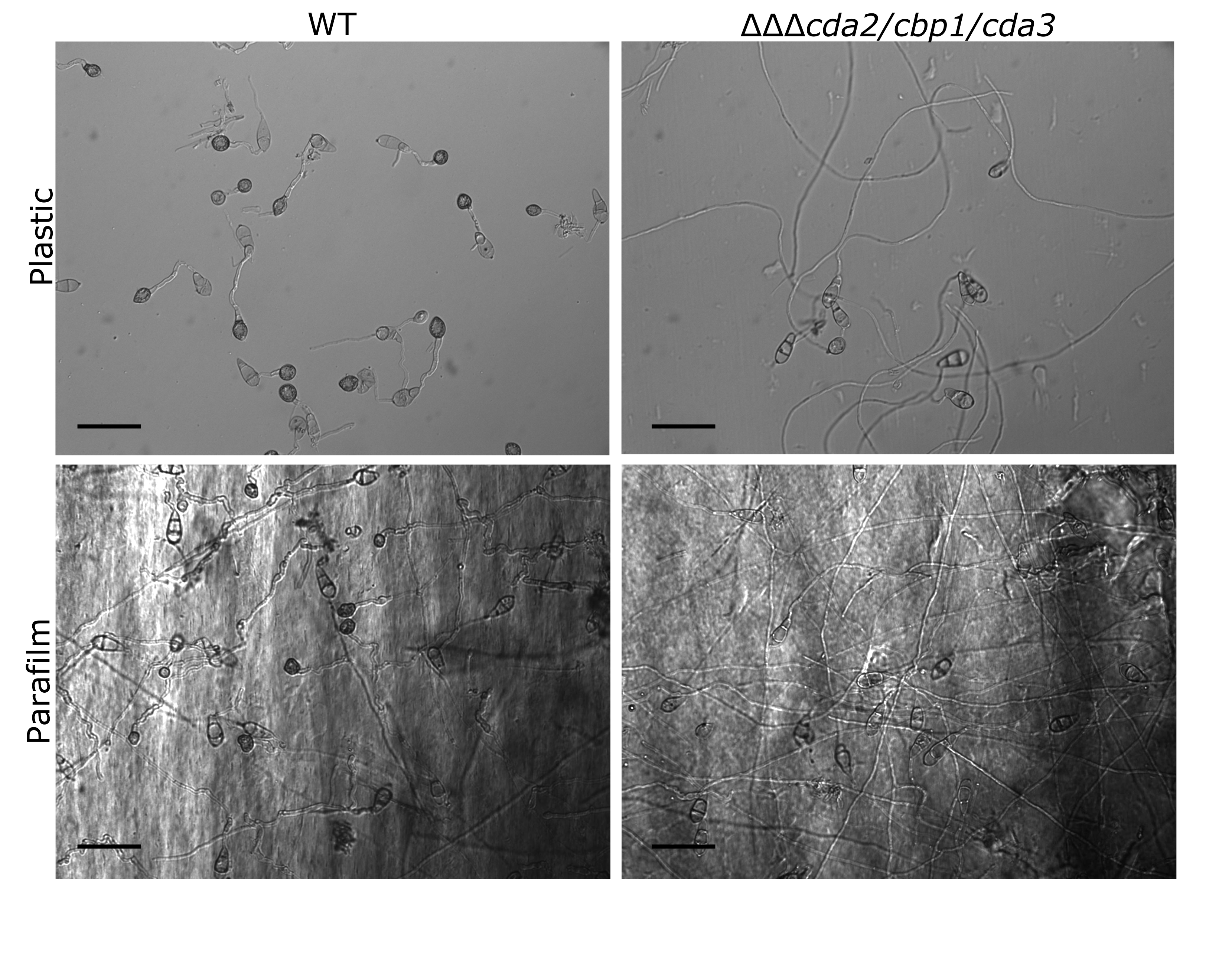

Supplement: S5 Fig — Conidia of the WT and cda2/cbp1/cda3 strains were inoculated onto the surfaces in 50 μl droplets, and incubated for 24 hr. Scale bars: 40 μm. (TIF) [file ppat.1005703.s005.tif]

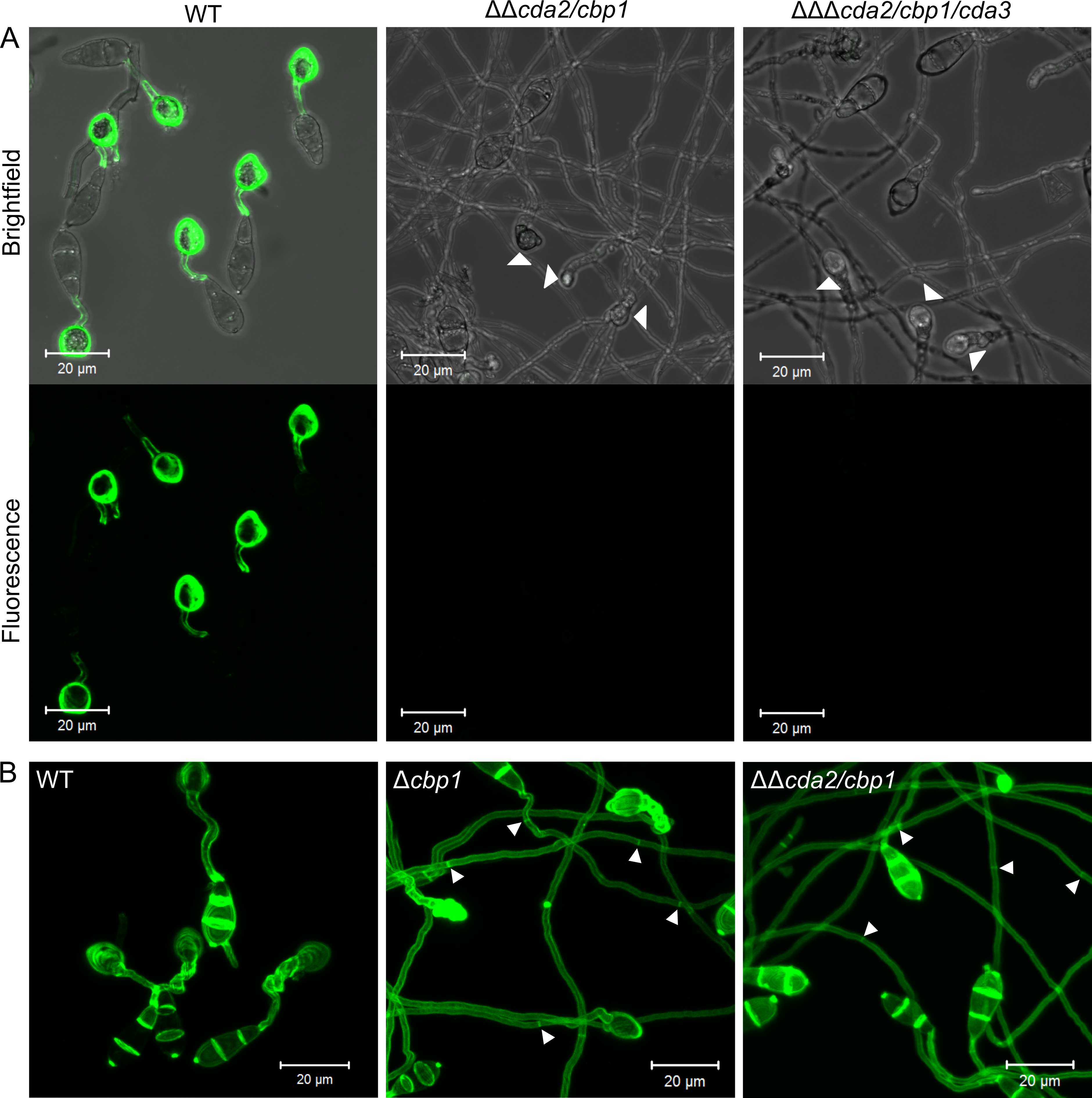

Supplement: S6 Fig — A) OGA488 staining of WT, cda2/cbp1 and cda2/cbp1/cda3 germlings at 24 hpi, showing absence of labelling in appressoria of the deletion strains (white arrow heads). B) Calcofluor white staining of germlings at 16 hpi, showing abnormally elongated, septate germ tubes (white arrow heads) in mutant strains, and abnormally shaped appressoria in cbp1. Scale bars: 20 μm. (TIF) [file ppat.1005703.s006.tif]

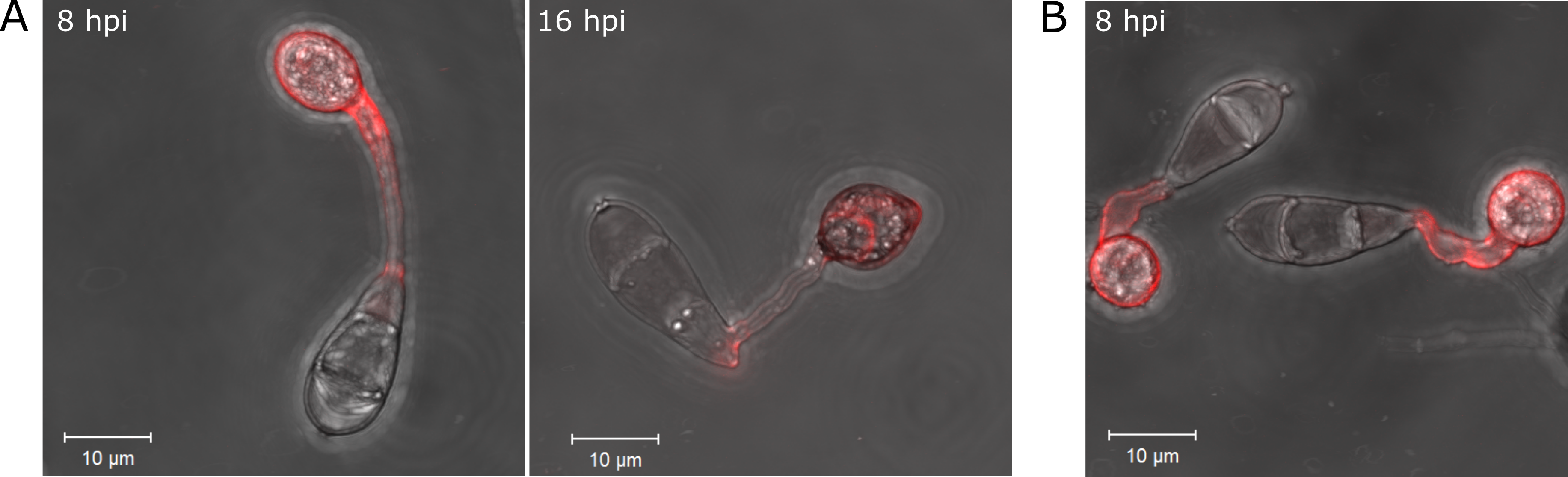

Supplement: S7 Fig — A) Cbp1:mCherry and B) Cda2:mCherry fluorescence at later stages of appressorium development (as indicated). Scale bars: 10 μm. (TIF) [file ppat.1005703.s007.tif]

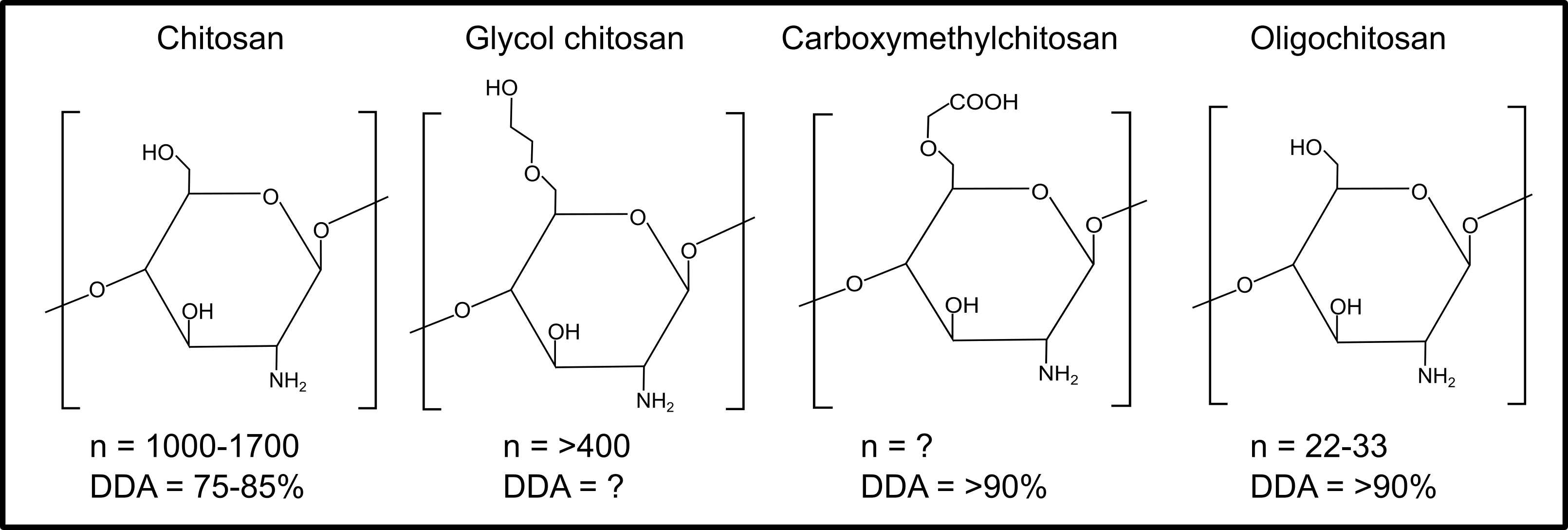

Supplement: S8 Fig — Molecular structure, degree of polymerization and degree of deacetylation (DDA) are indicated (when known). (TIF) [file ppat.1005703.s008.tif]

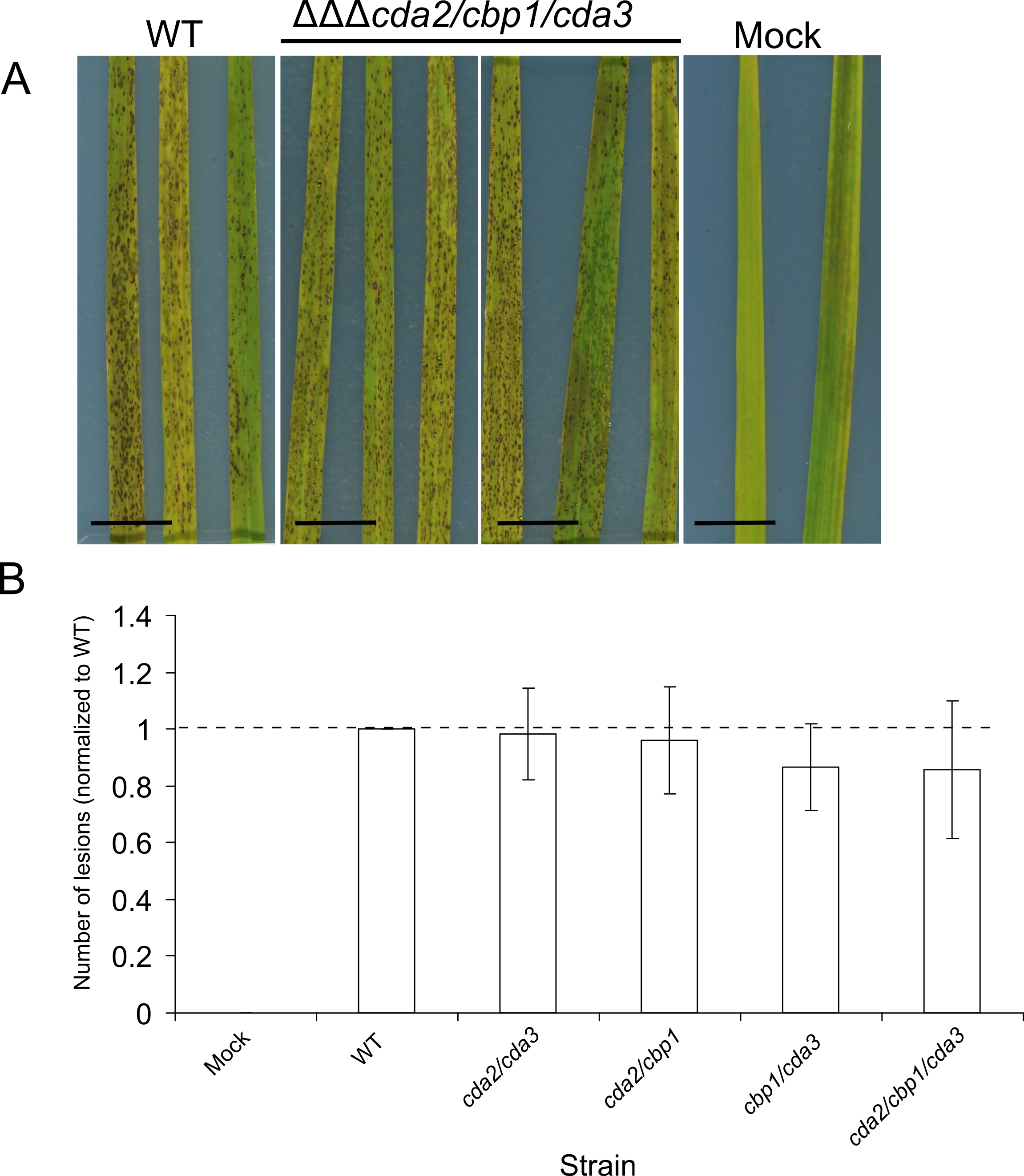

Supplement: S9 Fig — A) Pathogenicity of the cda2/cbp1/cda3 deletion strain on rice leaves, showing comparable lesion density to the WT strain (two independent triple deletion lines are shown). A mock inoculation of 0.2% gelatine was included as a negative control. Scale bars: 1 cm. B) Quantification of lesion numbers on detached rice leaves inoculated with different cda mutant strains, normalized to the Guy11 WT strain (± SD, n = 3). (TIF) [file ppat.1005703.s009.tif]

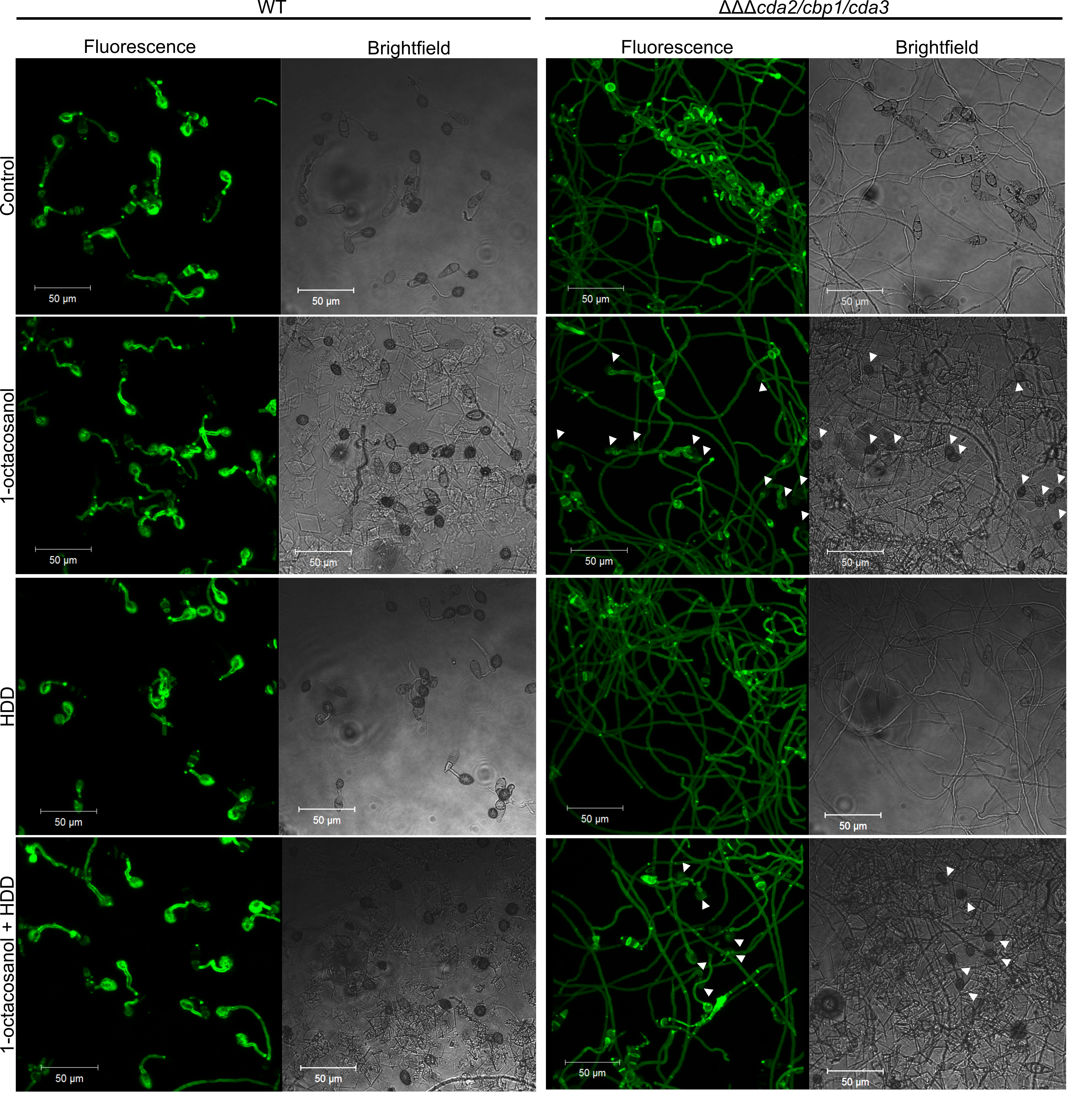

Supplement: S10 Fig — Appressorium development was observed in cda2/cbp1/cda3 (white arrow heads) in the presence of 1-octacosanol, but germ tubes remained abnormally elongated. Scale bars: 50 μm. (TIF) [file ppat.1005703.s010.tif]

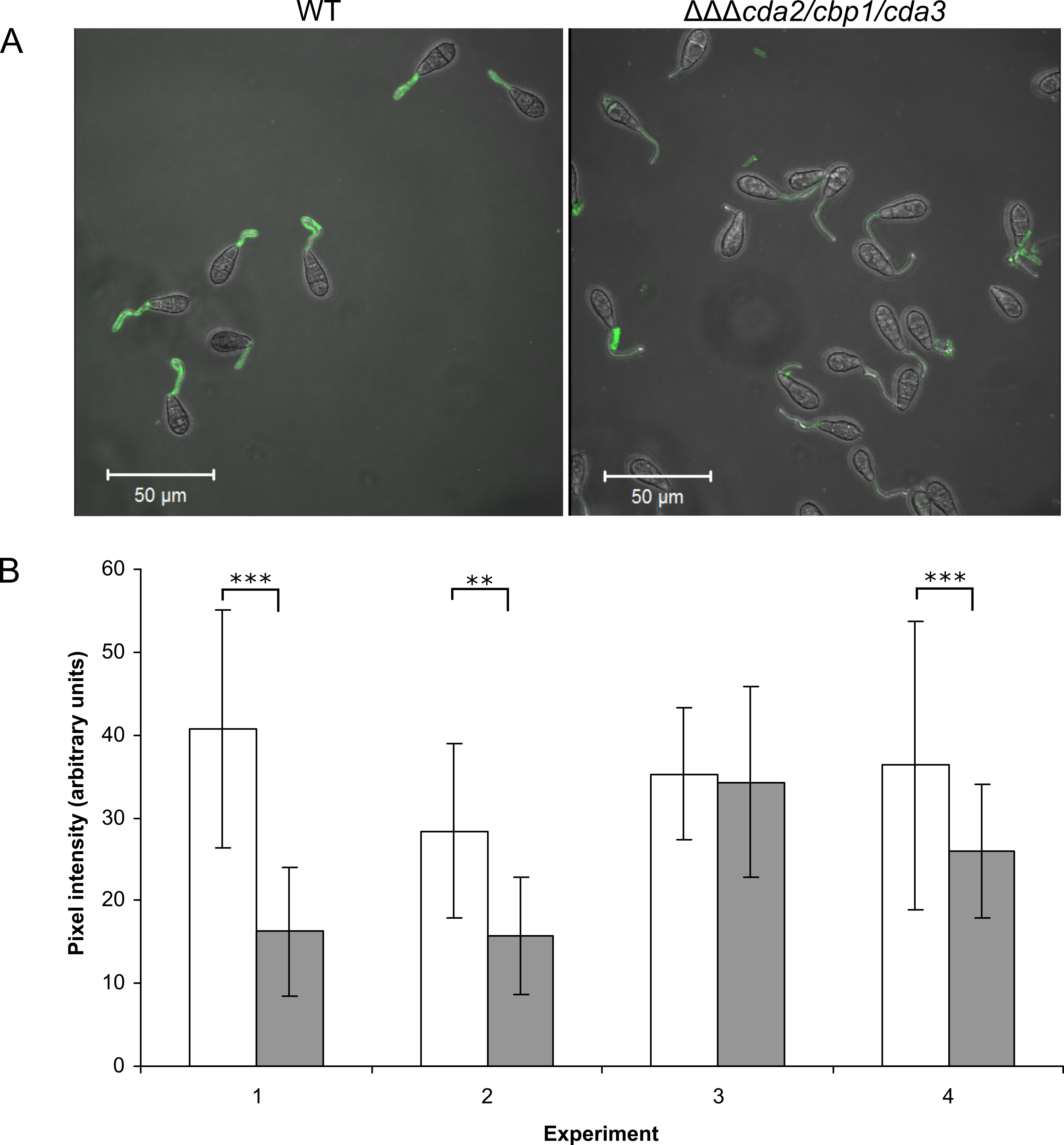

Supplement: S11 Fig — A) Germlings of the WT and cda2/cbp1/cda3 strain, stained with FITC-ConA. Pictures were taken from Experiment 1 (quantified in B). Scale bars: 50μm. B) Fluorescence intensity of germlings stained with FITC-ConA. Fluorescence intensities of ~140 germ tubes were measured, across 4 independent experiments. White bars: WT, grey bars: cda2/cbp1/cda3. Significant differences were found in 3 of the 4 experiments (2-way ANOVA with post-hoc Tukey test (** = p <0.01, *** = p < 0.001)) (TIF) [file ppat.1005703.s011.tif]

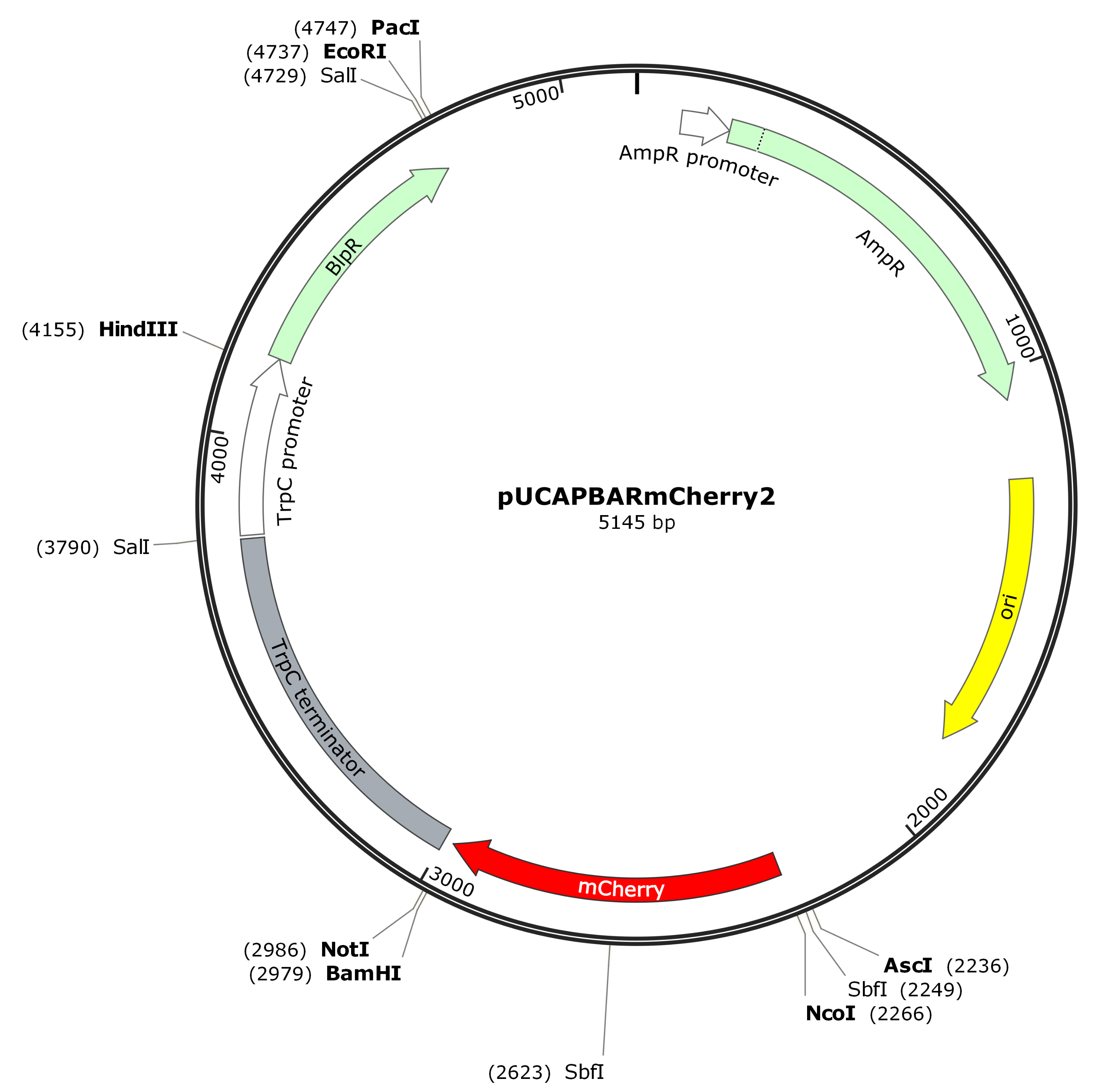

Supplement: S12 Fig — (TIF) [file ppat.1005703.s012.tif]

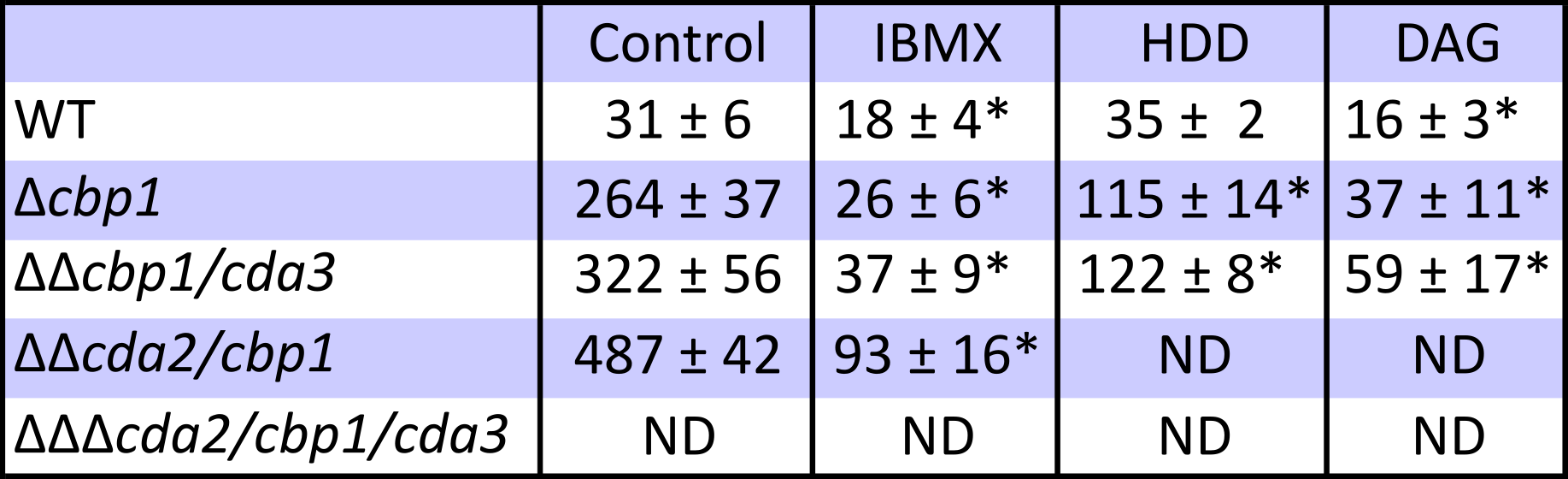

Supplement: S3 Table — Conidia of the WT and cda mutants were inoculated onto an artificial surface and incubated for 16 hr, in the presence of IBMX (3-isobutyl-1-methylxanthine), HDD (1,16 hexadecanediol; cutin monomer) or DAG (1,2-dioctanoyl-sn-glycerol). Significant (Mann-Whitney U-test, p < 0.001) reductions (asterisks) in germ tube lengths were observed in the deletion strains with all treatments, when compared with the control. ND—Not determined. (TIF) [file ppat.1005703.s015.tif]
